# DAT and TH expression marks human Parkinson’s disease in peripheral immune cells

**DOI:** 10.1038/s41531-022-00333-8

**Published:** 2022-06-07

**Authors:** Adithya Gopinath, Phillip Mackie, Basil Hashimi, Anna Marie Buchanan, Aidan R. Smith, Rachel Bouchard, Gerry Shaw, Martin Badov, Leila Saadatpour, Aryn Gittis, Adolfo Ramirez-Zamora, Michael S. Okun, Wolfgang J. Streit, Parastoo Hashemi, Habibeh Khoshbouei

**Affiliations:** 1 Department of Neuroscience, University of Florida, Gainesville, FL USA; 2 University of South Carolina, Columbia, SC USA; 3 Carnegie Mellon University, Pittsburgh, PA USA; 4 EnCor Biotechnology, Inc, Gainesville, FL USA; 5 Department of Neurology, University of Texas Health Science Center at San Antonio, San Antonio, TX USA; 6 Department of Neurology, Norman Fixel Institute for Neurological Diseases, UF Health, Gainesville, FL USA; 7 Department of Bioengineering, Imperial College, London, UK

**Keywords:** Neuroimmunology, Parkinson's disease, Neuroimmunology, Diagnostic markers

## Abstract

Parkinson’s disease (PD) is marked by a loss of dopamine neurons, decreased dopamine transporter (DAT) and tyrosine hydroxylase (TH) expression. However, this validation approach cannot be used for diagnostic, drug effectiveness or investigational purposes in human patients because midbrain tissue is accessible postmortem. PD pathology affects both the central nervous and peripheral immune systems. Therefore, we immunophenotyped blood samples of PD patients for the presence of myeloid derived suppressor cells (MDSCs) and discovered that DAT^+^/TH^+^ monocytic MDSCs, but not granulocytic MDSCs are increased, suggesting a targeted immune response to PD. Because in peripheral immune cells DAT activity underlies an immune suppressive mechanism, we investigated whether expression levels of DAT and TH in the peripheral immune cells marks PD. We found drug naïve PD patients exhibit differential DAT^+^/TH^+^ expression in peripheral blood mononuclear cells (PBMCs) compared to aged/sex matched healthy subjects. While total PBMCs are not different between the groups, the percentage of DAT^+^/TH^+^ PBMCs was significantly higher in drug naïve PD patients compared to healthy controls irrespective of age, gender, disease duration, disease severity or treatment type. Importantly, treatment for PD negatively modulates DAT^+^/TH^+^ expressing PBMCs. Neither total nor the percentage of DAT^+^/TH^+^ PBMCs were altered in the Alzheimer’s disease cohort. The mechanistic underpinning of this discovery in human PD was revealed when these findings were recapitulated in animal models of PD. The reverse translational experimental strategy revealed that alterations in dopaminergic markers in peripheral immune cells are due to the disease associated changes in the CNS. Our study demonstrates that the dopaminergic machinery on peripheral immune cells displays an association with human PD, with exciting implications in facilitating diagnosis and investigation of human PD pathophysiology.

## Introduction

Parkinson’s disease (PD) is marked by degeneration of substantia nigra (SN) dopamine neurons and reductions in central nervous system (CNS) dopamine^1–6^. This loss carries severe motor and non-motor symptoms^7–11^. In human and animal models, PD is validated in the CNS via markers of cell loss such as the dopamine transporter (DAT) and tyrosine hydroxylase (TH). But disease associated changes in dopaminergic markers, specifically DAT and TH, in peripheral immune cells have not been thoroughly studied. PD-associated changes that occur in the periphery have gained increasing interest in recent years. Although the brain-first versus periphery-first debate in PD is ongoing, the consensus in the field is that PD manifests in both CNS and peripheral compartments supporting the concept that the immune system, inflammation and compensatory anti-inflammatory responses play critical roles in PD^12–23^. T-cells, a component of the adaptive immune system, have been characterized extensively in PD. Experimental and clinical evidence have shown that CNS inflammatory processes in PD are associated with altered T-cell distributions and function^24–31^, with both CD4^+^ and CD8^+^ cells found near midbrain dopamine neurons^32–36^. Follow up studies by Kustrimovic et al. suggested that dopamine receptor expression on peripheral CD4^+^ T-cells are upregulated in PD and are positively associated with disease progression^37–39^, lending credence to immunological dysregulation of dopamine signaling in PD. Taken together, the peripheral immune system plays a critical role in PD^34,36,40–52^. Since innate immunity operates in concert with the adaptive immune system, we designed this study to complement the existing literature by focusing on peripheral blood mononuclear cells (PBMCs), specifically monocytes and other myeloid cells in PD patients compared to aged/sex matched healthy subjects.

Monocytes serve as front-line effector cells, and depending on the disease or injury, monocytes can adopt a range of intermediate phenotypes from pro-inflammatory to anti-inflammatory^53,54^. Importantly, PBMC-derived monocytes express an entire complement of dopaminergic signaling machinery: TH, vesicular monoamine transporter (VMAT2), dopamine receptors (DRD1-DRD5) and dopamine transporters (DATs)^20,47,55–62^. Immune cells’ dopamine receptor expression and function have been implicated in PD and PD-like neurodegeneration^63–66^. Previous reports show that monocytic TH expression serves an anti-inflammatory role^20,67,68^, and that expression of functional DAT on immune cells can attenuate inflammatory responses^69^. This suggests that dopaminergic proteins may mediate immune functions in PBMCs but whether PBMCs expressing DAT and TH delineate PD in human patients relative to healthy controls is unknown. To address this knowledge gap, we first immunophenotyped human PBMCs in healthy subjects and PD patients. Immunophenotyping of PBMCs derived from whole blood of 12 drug naïve (newly diagnosed) PD patients, 84 PD patients receiving treatment, and 62 healthy control subjects, showed that PD patients exhibited increased CD14^+^ monocytes, indicative of a pro-inflammatory state. Importantly, chronic inflammatory conditions are associated with a compensatory immunosuppressive phenotype, because, the immune system attempts to maintain homeostasis and control inflammation^70–73^. As such, we found PD patients exhibit increased immunosuppressive monocytic myeloid derived suppressor cells (M-MDSCs), with no change in other MDSC populations. These findings show that in PD, both inflammation and a targeted immune response occurs. We next applied a previously established^21^ flow cytometry protocol to quantify TH and DAT expressing PBMCs. We found DAT^+^/TH^+^ PBMCs were significantly *elevated* in PD. Additional experiments revealed that the readout of increased DAT^+^/TH^+^ PBMCs is not a generalized response of neurodegeneration, and it is likely a PD-specific component. While interesting, these data, on their own, do not provide mechanistic insight on whether reduced CNS dopamine in human PD is the causal mechanism for increased PBMCs expressing dopaminergic markers. Since mechanistic studies in human subjects are not feasible, we employed a reverse translational experimental design to investigate whether changes in peripheral immune cells’ dopaminergic markers in PD mice are due to loss of dopamine neuron. Our results show that following dopamine neuronal degeneration in the mouse CNS, the biochemical dopaminergic machinery on peripheral immune cells is altered, revealing a key mechanistic link. While our data do not illustrate the route by which these changes propagate to the peripheral immune system, the analysis of dopamine proteins in peripheral immune cells may offer novel insights into pathophysiology human PD.

## Results

### Parkinson’s patients’ PBMCs are polarized towards DAT^+^/TH^+^ monocytic-myeloid derived suppressor cells (M-MDSCs), suggesting a targeted immune response to PD

**Fig. 1:**
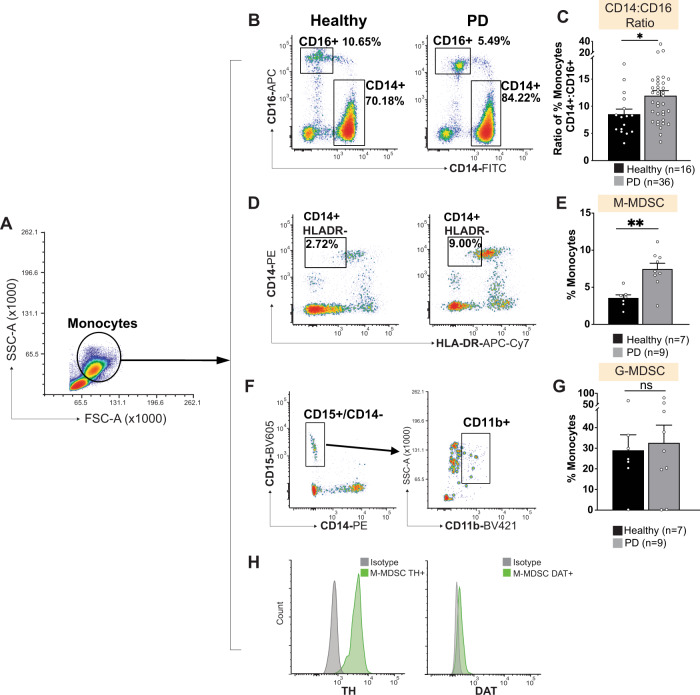
Parkinson’s patient monocytes are polarized towards a CD14+ pro-inflammatory phenotype with functional polarization towards monocytic-myeloid derived suppressor cells (M-MDSCs). Monocytes gated by scatter (**A**) were first analyzed for expression of CD14 and CD16 (**B**). Analysis confirms significant polarization of Parkinson’s patient monocytes towards the classical CD14+ monocyte phenotype (**C**); two-tailed *t*-test, *p* < 0.05. **D**, **E** Further immunophenotyping of Parkinson’s patient monocytes revealed a significant increase in the percentage of M-MDSCs characterized by expression of CD14 and absence of HLA-DR (**E**–**G**), relative to healthy controls. No change was found in granulocytic MDSCs (G- MDSCs) (**F**, **G**) characterized by expression of CD15 and CD11b. **H** M-MDSCs express both DAT and TH. Routine metrics such as age distribution, sex distribution, disease duration, treatment, LED score and motor scores, along with statistical analysis are included in Supplementary Tables 1 and 2. All data shown are ±SEM.

PD patients exhibit peripheral and central nervous system inflammation^74–83^, but there is little information about peripheral immune response to the inflammatory state. Peripheral blood mononuclear cells (PBMCs) serve as front-line effector cells and are sensitive to inflammation^84,85^. Therefore, we investigated whether PD patients’ PBMCs reflect the CNS and/or peripheral immune response to the inflammatory state. The PBMC fraction contains multiple monocyte subsets with varied functions. Functional properties of monocytes are classified according to the expression of cell surface markers^86–88^. Historically, myeloid cells, including monocytes and brain-resident microglia, are grouped into inflammatory and resting states, i.e., M1 and M2 respectively^89^. This categorization is now recognized to be overly simplistic^90–94^, but still serves as a useful basic step when followed by more-nuanced immune characterization^86,94–96^. Classification scheme for monocytes uses the surface marker CD14 to characterize inflammatory monocytes, whereas CD16 surface marker characterizes resting monocytes^53^. A bias towards one or the other monocyte subset (CD14 vs. CD16) would suggest a pro- or anti-inflammatory state. For example, a higher ratio of CD14 to CD16 suggests an inflammatory state^53,97–99^. It should be noted that inflammation and the subsequent adaptive anti-inflammatory response can occur concurrently^70–73^. Therefore, we assessed monocyte polarization towards pro- or anti-inflammatory (CD14 or CD16) states in healthy subjects and PD patients. We observed a distinct polarization towards the pro-inflammatory CD14^+^ phenotype in PD patients (Fig. 1B, C). No differences were found in intermediate monocytes (CD14^+^CD16^+^, Supplementary Fig. 2) between PD patients and control group. Consistent with the literature, increased CD14:CD16 ratio in PD indicates a state of persistent inflammation, that is associated with immunosuppressive response(s). Most notably, myeloid derived suppressor cells (MDSCs) are a subset of CD14^+^ monocytes which inhibit inflammation^41,86,96,100–104^. It has been shown that MDSCs arise as a compensation to chronic inflammation^105–108^ supporting the hypothesis that the elevated CD14:CD16 ratio is associated with increased immunosuppressive MDSCs in PD patients. MDSCs are traditionally categorized as monocytic (M-MDSCs) or granulocytic (G-MDSCs)^109,110^. Therefore, we immunophenotyped MDSCs into M-MDSCs (CD14^+^ HLA-DR-neg) or G-MDSCs (CD14-neg CD15^+^ CD11b^+^)^86,110–113^. Consistent with data shown in Fig. 1B, C, we found a significant increase in M-MDSCs with no change in G-MDSCs (Fig. 1F, G), suggesting that specific immune adaptations occur in PD^114–119^.

In myeloid cells, dopaminergic proteins such as DAT and TH modulate immune functions and attenuate runaway inflammation^20,67–69,120–123^. For instance, DAT activity on peripheral immune cells regulates cytokine release following immune stimulation^123^. A higher percentage of DAT^+^/TH + PBMCs could be an indication of a targeted immune response in PD^69,123^. This interpretation is consistent with previous reports suggesting TH and DAT expression are associated with an immunosuppressive phenotype^67,68,71,72,110,124^. Therefore, next we investigated whether increased M-MDSCs in PD patients is accompanied by TH and DAT expression. We found that *all* M-MDSCs are DAT^+^/TH^+^ (Fig. 1H). Consistent with recent reports^20,67,68^, these data support the interpretation that dopaminergic proteins may mediate immune functions in PBMCs, and that PBMCs expressing DAT and TH may delineate PD in human patients relative to healthy controls.

### Increased TH- and DAT-expressing PBMCs mark peripheral immune cells of PD patients

**Fig. 2:**
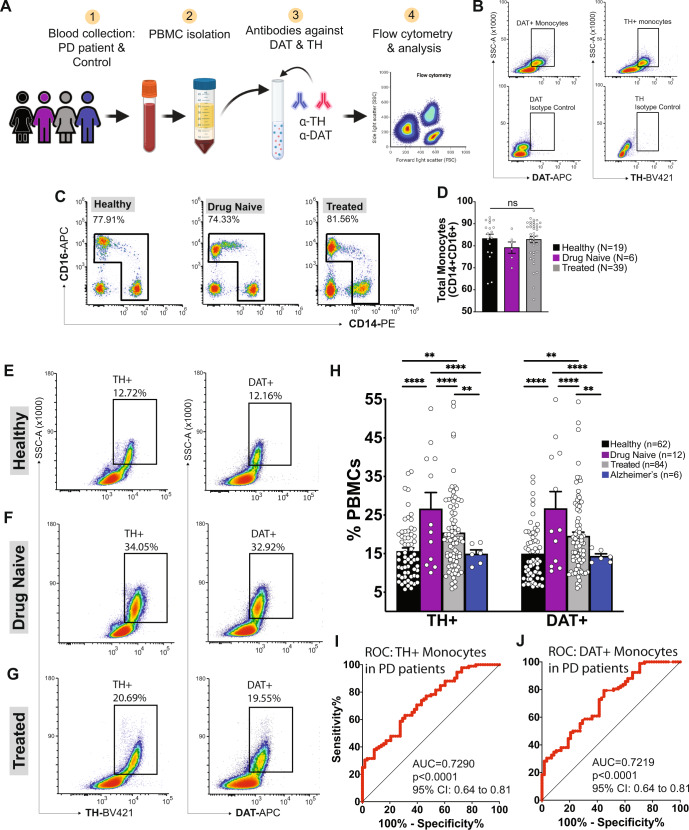
Parkinson’s disease patients exhibit an increase in PBMCs expressing tyrosine hydroxylase (TH) and dopamine transporter (DAT). **A** Schematic of workflow. **B** Representative flow cytometry plots for DAT and TH shown in healthy subjects (top row). Staining with isotype control antibodies (bottom) shows minimal background. **C**, **D** Total monocytes (all CD14+ and CD16+ cells) were analyzed in healthy controls, drug naïve, and treated PD patients, revealing no change in total monocytes among these groups. **E**–**G** Monocytes analyzed for expression of TH and DAT reveals that drug naïve Parkinson’s patients exhibit a significant increase in TH+ and DAT+ monocytes (**F**). Following treatment, levels approach those in healthy controls (**G**), but both drug naïve (*n* = 12) and drug treated Parkinson’s disease patients (*n* = 84) exhibit significantly increased expression in DAT and TH relative to healthy controls (*n* = 62). Patients with Alzheimer’s Disease (*n* = 6) show no increase in DAT and TH expressing cells (**H**) (one-way ANOVA with Tukey’s correction, alpha = 0.05). Data shown are ±SEM. **I**, **J** Receiver operating characteristic (ROC) analysis reveals a significantly greater-than-chance accurate classification of Parkinson’s patients using DAT+ and TH+ PBMCs. Cutoffs were set at 13.55 and 12.99, respectively, by Youden’s index. Area under the curve (AUC) shown suggests significantly greater than chance (represented by diagonal black line) detection of Parkinson’s disease by TH+ and DAT+ PBMC analysis. Routine metrics such as age distribution, sex distribution, disease duration, treatment, LED score and motor scores, along with statistical analysis are included in Supplementary Tables 1 and 2.

Patients were recruited at the time of PD diagnosis (drug-naïve) and after initiating standard-of-care PD treatments (treated) (Fig. 2A). Drug-naïve PD patients were recruited at the time of diagnosis following evaluation by a movement disorders specialist, prior to initiation of treatment. Treated PD patients were recruited at standard-of-care visits and had been prescribed pharmacotherapies, shown in Supplementary Table 1 (i.e. L-DOPA, dopamine agonists), deep brain stimulation (DBS) or a combination of these. Healthy controls were either the patient’s caregiver/spouse or recruited as age-and sex-matched controls. Spouses were considered the optimal control group, despite different sex, due to similar diet, environmental factors, and age^125–128^. PD patients were compared to age/sex-matched control subjects. PBMCs from patients with Parkinson’s disease and healthy control subjects were isolated by density gradient centrifugation using Ficoll-Paque and assessed by flow cytometry to detect myeloid cells expressing TH and DAT^20,21,69,123^, (Fig. 2B, Supplementary Fig. 1). In accordance with increased CD14^+^ monocytes and increased MDSCs in PD, as a first step, we determined whether total monocytes (i.e., CD14 and/or CD16 monocytes) (Fig. 2C, D), differed between the experimental groups. Total monocytes in drug naïve PD patients, treated PD patients, and healthy controls were not significantly different from one another (One-way ANOVA, *F*(2,61) = 0.52, *p* = 0.60), indicating no differences in total monocytes (Fig. 2C). Also, no sex difference was observed (Supplementary Fig. 3). Multiple linear regression (*n* = 158) considering disease duration, age, treatments, and comorbid conditions showed no significant interaction (Supplementary Table 3).

Since immune cells express DAT and TH, and that CNS dopamine neurons expressing the same markers are decreased in PD patients, we next asked whether or not DAT^+^ and TH^+^ PBMCs were altered in PD patients compared to healthy controls, and whether disease duration, sex, age, treatments, and comorbid conditions affect these measures. Consistent with our hypothesis and in line with our recent report^20^, PD patients (both drug-naïve and treated) exhibit increased percentages of DAT^+^/TH^+^ PBMCs. Relative to healthy control group (Fig. 2E–H), drug-naïve PD patients show a significant increase in DAT^+^/TH^+^ PBMCs (Fig. 2F, H). PD patients treated with standard-of-care treatments show reduced DAT^+^/TH^+^ PBMCs relative to drug-naïve patients, but still significantly higher than controls (Fig. 2G, H) (One-way ANOVA with Tukey’s correction, *F*(3,4) = 317.1, *p* < 0.01, *p* < 0.001, *p* < 0.0001). Multiple linear regression considering disease duration, age, sex, treatments, and comorbid conditions showed no significant interaction (Supplementary Table 3). To determine whether the neuroinflammation which occurs in both AD^129–133^ and PD^77,134–137^ could produce a similarly increased percentage of DAT^+^/TH^+^ PBMCs that could be generalized to neurodegeneration, we repeated these experiments in a cohort of Alzheimer’s diseases (AD) patients with no movement disorder, psychoses, and no use of dopaminergic drugs (i.e. neuroleptics, antidepressants). DAT^+^/TH^+^ monocyte populations in AD patients are indistinguishable from healthy controls, leading us to conclude that increased numbers of monocytes expressing markers of dopamine signaling are not general to all neurodegenerative diseases. These results suggest that at the time of diagnosis, drug-naive PD patients exhibit significantly increased PBMCs co-expressing dopaminergic markers DAT and TH, and that treatment for PD may modulate peripheral immune cells expressing dopaminergic machinery. We also note that increased percentage of DAT^+^/TH^+^ PBMCs in PD, closely matches the percentage increase in DAT^+^/TH^+^ MDSCs in PD patients (Fig. 1). Due to methodological constraints involving antibody cross reactivity, we cannot confirm the likely hypothesis that the increased DAT^+^/TH^+^ PBMCs is entirely comprised of MDSCs. While flow cytometry detects DAT and TH expressing cells, it does not assess protein levels of these markers. Nevertheless, the absence of changes in the total number of monocytes in PD vs. healthy subjects suggests that increased DAT^+^ and TH^+^ PBMCs represent a phenotypic change. Lastly, a single patient in a cohort of 96 samples had three distinct DAT^+^ TH^+^ PBMC populations (instead of one). This patient was not a statistical outlier or distinguished by any other criteria such as disease duration, age, sex, treatments, and comorbid conditions, therefore providing no rationale for exclusion. While interesting, we are unable to comment on the potential immunological significance of this observation in one sample.

DAT bears homology to other biogenic amine transporters, such as the norepinephrine transporter (NET) and serotonin transporter (SERT)^138–142^. We and others have shown that myeloid cells in the PBMC fraction express NET but not SERT^69,123,143^. Therefore, we asked whether NET^+^ PBMCs are increased in PD in a similar manner to DAT^+^ PBMCs. We assessed NET^+^ PBMCs in a separate cohort of PD patients and healthy controls (Supplementary Fig. 4). In this separate cohort, we found that PD patients (*N* = 8) exhibited significant increases in DAT^+^ and TH^+^ PBMCs relative to healthy control subjects (*N* = 4) (t-test with Holm-Sidak correction, *P* < 0.05, *N* = 4–8 independent biological replicates per group), but NET^+^ PBMCs are not significantly different between groups (two-tailed *t*-test with Holm-Sidak correction, *p* = 0.802).

PD is more commonly diagnosed in males than in females^144–149^, and multiple research groups have investigated sex-specific factors in the etiology of PD. Therefore, we determined whether the measured increased DAT^+^/TH^+^ PBMCs in drug naïve and treated PD patients show sex differences. Considering sex as a biological variable, males and females were analyzed separately, and analyzed together in multiple linear regression considering disease duration, age, treatments, and comorbid conditions (Supplementary Table 3). No significant interactions were found, suggesting males and females do not exhibit significantly different percentages of DAT^+^/TH^+^ PBMCs (Supplementary Figure 5). It is however possible that the potential differences are not detectable in a cohort of 158 total samples.

Current PD diagnoses occur after onset of symptoms (where the majority of dopamine cells are lost) and are based on combined motor performance^10,150–153^ and drug response^10,154–156^, with ~70% accuracy^157^. There are no complementary blood tests to confirm PD diagnosis at the disease onset or serve as an early screening tool. We therefore asked whether increased DAT^+^/TH^+^ PBMCs could be adapted to serve as a complementary diagnostic tool. Receiver Operating Characteristic (ROC) analysis is routinely used to evaluate the accuracy of a new diagnostic test in comparison to an existing diagnostic test^158,159^. In ROC analysis, area-under-the curve (AUC) with a value of 1 indicates a perfect diagnostic test. A ROC curve with AUC = 0.7–0.8 indicates a test with good diagnostic potential. As shown in Fig. 2I, J, ROC analysis of PBMCs expressing DAT and TH, in patients diagnosed with PD by a movement disorders specialist (defined as ‘clinical truth’) were compared to control subjects. ROC analyses revealed that both TH^+^ PBMCs (AUC = 0.72) and DAT^+^ PBMCs (AUC = 0.72) exhibit a significantly greater than chance (*p* < 0.0001) predictive value in identifying PD patients even among a small of 96 subjects (Fig. 2 I, J). Importantly, AUCs for TH^+^ PBMCs and DAT^+^ PBMCs showed a predictive value similar to conventional PD diagnostics^157^. Additional analysis using Youden’s Index suggests diagnostic cut points at 12.99% and 13.55% for TH^+^ PBMCs and DAT^+^ PBMCs, respectively. ROC AUC was similar when data were separated into male and female cohorts (Supplementary Fig. 6), suggesting this metric is applicable to both sexes. Thus, this approach could be developed to complement existing diagnostics and has exciting implications for early PD diagnosis/screening.

### Dopamine depletion and replenishment mediates % DAT^+^ and TH^+^ PBMCs, further implicating the sensitivity of these markers for PD

To investigate the mechanistic underpinning of the data in human PD, we examined whether loss of CNS dopamine neurons impact peripheral myeloid cells expressing DAT and TH by employing a reverse-translation strategy in two complementary mouse models of PD. To model CNS dopamine neurodegeneration, we used intracranial 6-hydroxydopamine (6-OH-DA)^160,161^. This model produces an irreversible loss of CNS dopamine neurons and subsequent dopamine depletion in the substantial nigra^162–164^. To model dopamine depletion in the absence of true neurodegeneration, we used *i.p*. 1-methyl-4-phenyl-1,2,3,6-tetrahydropyridine (MPTP)^165–167^. In the MPTP model of PD, dopamine neurons transiently lose TH immunoreactivity^168–172^.

**Fig. 3:**
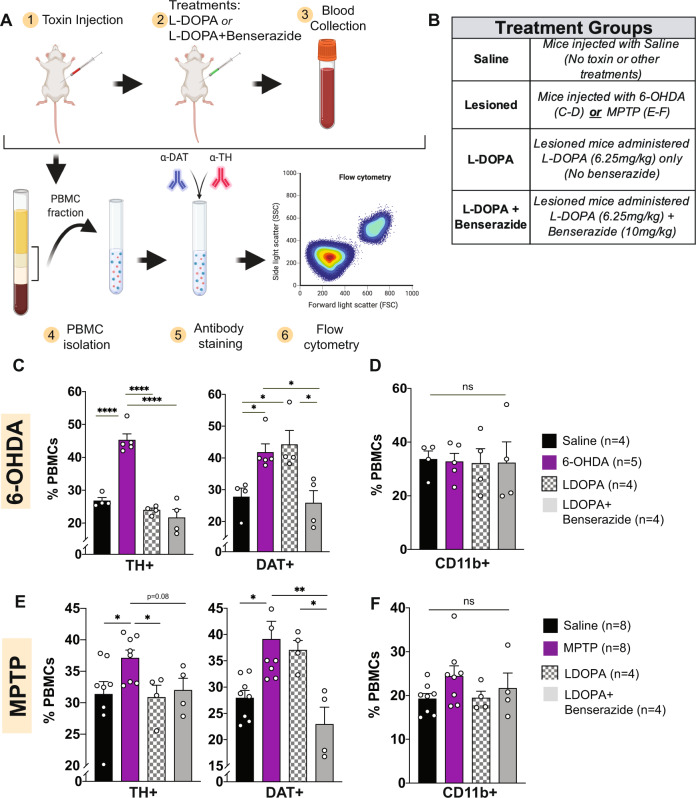
Depletion of dopamine neurons with 6-OHDA or MPTP in mice increases numbers of DAT^+^ and TH^+^ PBMCs and treatment with L-DOPA+ Benserazide restores baseline levels. Treatment with L-DOPA alone restored DAT^+^ but not TH^+^ PBMCs. **A** Schematic of experimental design using toxin-induced lesions. **B** Animals were grouped by treatment conditions as shown. **C**, **D** Bilateral infusion of 6-OHDA (5 mg/mL) into the median forebrain bundle, or acute depletion by four i.p. MPTP injections (**E**, **F**) reveal that PD-like lesions in mice reproduce the phenomena observed in Parkinson’s patients and healthy controls. **C**, **E** Both TH+ and DAT+ cells are increased following dopamine neuron depletion with 6-OHDA (**C**) or MPTP (**E**). TH+ and DAT+ cells are restored to, or trend towards, healthy levels following treatment with L-DOPA+ AADC inhibitor Benserazide. In both PD models, TH-expressing cells are reduced to near-healthy levels following LDOPA treatment alone, while DAT-expressing cells are reduced to healthy-levels only with L-DOPA+ Benserazide, suggesting separate regulatory steps for DAT and TH expression in peripheral immune cells. **D**, **F** Total myeloid cells, defined by CD11b expression, are not changed amongst treatment groups in either model. All data shown are ±SEM.

We interrogated whether brain dopamine depletion (MPTP model) or dopamine neuron death (6-OH-DA model), recapitulate the altered DAT^+^/TH^+^ PBMCs in human PD. Consistent with the literature and as shown in Supplementary Fig. 5C, intracranial 6-OHDA lead to >60% loss of dopamine neurons in the substantia nigra. PBMCs isolated from whole blood were assayed for expression of CD11b (pan-monocyte marker), DAT and TH expression. Similar to PD patients (Fig. 2), we found that 6-OHDA, but not saline treated mice, exhibited an increased percentage of DAT^+^/TH^+^ PBMCs, with no change in total CD11b^+^ monocytes (Fig. 3C, D). These data provide a mechanistic link between loss of CNS dopamine neurons and changes in dopaminergic markers on the peripheral immune cell. While multiple potential pathways and signaling molecules are likely involved in CNS-to-periphery communication, these data reveal the overall consequences of CNS dopamine depletion on peripheral immunity. We used the MPTP mouse model of PD to test the possibility that dopamine depletion alone, in the absence of neuronal loss, is sufficient to trigger peripheral immune changes. Methodological details and sampling timeline are described in Methods. We found MPTP-mediated dopamine depletion also increased the percentage DAT^+^/TH^+^ PBMCs (Fig. 3E, F), suggesting that reduction of CNS dopamine, can affect dopaminergic markers on the peripheral immune cell. Collectively, our data support the interpretation that both neuronal loss and dopamine depletion recapitulate PD patients’ data. These results also provide a mechanistic link between loss of CNS dopamine which leads to increased DAT^+^/TH^+^ PBMCs in human PD, introducing a direct readout of human PD as a non-invasive and accessible liquid biopsy.

Given that increased DAT^+^/TH^+^ PBMCs are conserved between human and the animal PD models used in this study, and that dopamine depletion clearly underlies this response, we next asked whether the changes in DAT^+^/TH^+^ PBMCs are sensitive to dopamine replenishment. In Fig. 2, we showed that in therapeutically treated PD patients, the percentage of DAT^+^/TH^+^ PBMCs decline towards healthy levels. Almost all PD treatments increase CNS dopamine. Using a reverse translation strategy, we tested the hypothesis that in PD mice an analogous pharmacological approach such as L-DOPA+ carbidopa/benserazide administration^173–183^ would lower PBMCs expressing DAT and TH towards sham control levels. We found, PD mice receiving five days of i.p. L-DOPA+ Benserazide^56,156^ show reduced percentage of PBMCs expressing DAT and TH (Fig. 3C, E) as compared to saline-treated PD mice. These results obtained via a complementary approach further confirm: (1) there is a communication between CNS and peripheral immune cells, (2) CNS dopamine depletion underlies increased DAT^+^/TH^+^ PBMCs, and importantly, (3) treatment targeted towards replenishing brain dopamine restores peripheral DAT^+^/TH^+^ PBMCs, further validating the sensitivity of these markers for PD.

Since L-DOPA+ Benserazide increases dopamine in CNS and periphery, we investigated whether restoring dopamine only in the periphery produces a similar outcome. In both PD mouse models, animals received L-DOPA alone (L-DOPA metabolizes in the periphery, thus does not reach the brain) or L-DOPA+ Benserazide (benserazide inhibits peripheral metabolism of L-DOPA, allowing it to reach the brain), which increases both CNS and peripheral dopamine levels. In PD mice, five days of L-DOPA administration did not restore the % of DAT^+^ PBMCs to pre-PD/saline control. In contrast, TH^+^ PBMCs were restored to pre-PD levels following L-DOPA administration alone in both animal models (Fig. 3C, E), suggesting in mice monocyte expression of TH and DAT are regulated via separate mechanisms, therefore exhibiting different patterns of expression following systemic administration of L-DOPA alone. While these data are intriguing, due to patient safety and ethical restrictions in providing substandard clinical care, we do not have access to PD patient cohort receiving L-DOPA alone to investigate whether or not a similar modulation of TH^+^ PBMCs occurs in human PD. However, the transformative observation that DAT and TH expression levels on the peripheral immune system in PD show a great promise as non-invasive readouts.

Lastly, we investigated whether, similar to PD patients, PD mice exhibit increased M-MDSCs. Ly6C and Ly6G are analogous to human M-MDSCs and G-MDSCs respectively. Neither 6-OHDA or MPTP PD model exhibited an increase in M-MDSCs, while MPTP mice showed a trend towards decreased G-MDSCs (Supplementary Fig. 9, C, D). Given that M-MDSCs arise over a protracted time course^105,106,184,185^, the short duration of PD mouse lesions may be insufficient to induce an MDSC phenotype. Our ongoing longitudinal studies investigating progressive changes in M-MDSCs in the PD patients will reveal the time course of peripheral immunological consequences in PD.

## Discussion

Our data are in line with published reports of anatomic connections between peripheral immunity and dopamine neurons as well as brain to periphery in general^50,186–188^, including compensatory immunological responses following CNS dopamine depletion. More than two decades of research support the interpretation that in PD, dopamine neurodegeneration produces inflammatory responses, involving multiple inflammatory mediators and various cell types^37,189,190^. For example, microglia express dopamine receptors. Dopamine activation of microglial dopamine receptors regulates the release of inflammatory mediators^47,191,192^. In disease states with CNS dopamine depletion, the levels of inflammatory mediators are dysregulated^37,189,190^. In PD, there is a strong association between CNS inflammation and peripheral inflammation, but the source of increased peripheral inflammation and inflammatory molecules in the periphery are unclear. Dopamine neuronal loss can theoretically lead to a runaway inflammatory response that affects peripheral immune responses, which in turn may exacerbate CNS inflammation. Multiple groups have shown that dopaminergic protein expression and modulation on peripheral immune cells can impact PD and PD-like degeneration^37–39,63–66^. The identified increased DAT^+^/TH^+^ PBMCs, plus our discovery of increased MDSCs, suggests these immunological changes are responses to an inflammatory state in PD. While we acknowledge a limitation of our study in providing mechanistic relationships between DAT^+^/TH^+^ PBMCs and immunosuppression, extensive literature supports the notion that dopaminergic machinery on immune cells suppresses inflammatory immune functions^20,47,60–66,69,120,123,193,194^. Overall, our data support the interpretation that CNS dopamine depletion modulates peripheral immunity and expression of dopaminergic markers DAT and TH on peripheral immune cells.

PD is a chronic disease that develops over decades, and patients experience a plethora of symptoms directly and indirectly related to brain dopamine depletion. To identify the immunological consequences of dopamine depletion in PD, we employed a reverse translational strategy in two animal models of PD. Our results show that peripheral immune cells are responsive to CNS dopamine neuronal depletion. However, our data do not illustrate the route by which these changes propagate to the peripheral immune system. The CNS originating inflammatory factors, capable of influencing the peripheral immune system, are released from CNS to the peripheral circulation, via multiple routes^195–198^. Three prevailing hypotheses describe mechanisms by which CNS cytokines impact peripheral immune cells: (1) a selective permeabilization of the blood-brain-barrier in specific brain regions allows inflammatory factors to enter peripheral circulation^195,196^, (2) molecules originating in the brain exit the CNS parenchyma into cerebrospinal fluid, making their way to the venous bloodstream via subarachnoid granulations^197,198^ and (3) deep brain structures, such as the basal ganglia, secrete inflammatory mediators into glymphatic vessels during neuroinflammation, which drain into cervical lymph nodes and eventually emerge into the peripheral circulation^199–201^. The characterization of potential routes by which CNS-originating factors in PD enter the periphery, whether by blood-brain-barrier permeabilization, drainage via subarachnoid granulations or through the glymphatic system, represents a decade or more of mechanistic studies that is beyond the scope of this investigation. Most critical, however, is that the analysis of dopamine proteins in peripheral immune cells may offer novel and practical diagnostic and/or prognostic insights into human PD.

## Methods

### Study design

The aim of this study was to determine whether dopaminergic markers DAT and TH were altered on peripheral immune cells of PD patients. To investigate whether DAT and TH expression are altered in PD, we recruited PD patients, healthy control subjects and Alzheimer’s disease patients as a control cohort. Criteria are described below. PBMCs from all cohorts were analyzed via previously published flow cytometry protocols. We further confirmed these results using two distinct animal models of PD-like dopamine depletion and dopamine neuron degeneration. Required sample sizes for human and mouse cohorts were determined by power analyses using preliminary data, with alpha = 0.05 and a power of 80% for group comparison. Sample preparation, data collection and data analysis were all performed by blinded investigators. No outliers had to be considered. Numbers of experimental replicates are described in figure legends as appropriate.

All human subjects research was carried out in accordance with University of Florida IRB, as described below. Other than rodent euthanasia criteria as specified by University of Florida IACUC and University of South Carolina IACUC, no endpoints needed to be specified. All animal studies were conducted in accordance with University of Florida and University of South Carolina, Columbia IACUC. Detailed information regarding ethics are provided in Methods below.

### Human subjects

Blood samples from age-matched healthy subjects were obtained from two sources between August 2017 and January 2020: the Movement Disorder Clinic at the University of Florida *via* an approved IRB protocol with written informed consent including consent to publish indirect identifiers (IRB201701195), or the Lifesouth Community Blood Center, Gainesville, FL where deidentified samples exempt from informed consent (IRB201700339) were purchased. According to Lifesouth regulations, healthy donors were individuals aged 50–80 years-old of any gender, who were not known to have any blood borne pathogens (both self-reported and independently verified), and were never diagnosed with a blood disease, such as leukemia or bleeding disorders. In addition, none of the donors were using blood thinners or antibiotics, or were exhibiting signs/symptoms of infectious disease, or had a positive test for viral infection in the previous 21 days.

### Inclusion/exclusion criteria for human subjects

#### Parkinson’s disease patients

Potential study participants were evaluated by a board-certified neurologist specializing in movement disorders. Patients were eligible to participate if (1) they had a confirmed PD diagnosis, (2) there was absence of comorbid movement disorder (i.e. essential tremor), (3) there was absence of any psychiatric diagnoses, (4) they were not prescribed psychotropic medications (i.e. neuroleptics), (5) they had no current or recent diagnosis of cancer (within 18 months) and were not on current or recent (within 18 months) treatment for the same, (6) had not been diagnosed with viral, bacterial or other infections within the preceding 21 days and were currently not being treated for the same.

#### Alzheimer’s disease patients

Potential participants were evaluated by a board-certified neurologist specializing in dementias. Participants were eligible to enroll if they (1) had a positive diagnosis of Alzheimer’s disease, (2) in the absence of any motor disorder, and (3) were not currently using medications which may impact dopaminergic function (i.e. antipsychotics, neuroleptics, dopaminergic antidepressants).

#### Healthy control subjects

While not evaluated explicitly by a movement disorder specialist, healthy control subjects were present at the time of blood draw for PD patients and most frequently included the patient’s spouse, allowing for control of environmental factors that may influence immune factors being studied. Participants were eligible to participate if (1) they report no current or past diagnosis of motor disorder (PD, ET, dystonia), (2) they were currently not taking medication for the same (self-reported), and (3) were not exhibiting overt symptoms of movement disorder.

#### Demographic information

Data on each subject included in the study are presented in Supplementary Table 1. Age, disease duration, sex distribution and motor scales for groups used for analysis in each figure panel are given in Supplementary Table 2.

### Animals

Animal studies were performed in compliance with guidelines from the National Institutes of Health and with guidelines from University of Florida IACUC (IACUC#202008450), and University of South Carolina IACUC (IACUC#2459-101447-070819). Adult male mice (>56 days old) on a C57BL/6 background were used for experiments. After 6-OH-DA surgery or MPTP injection, animals were provided with dishes of diet gel (Clear2O, 76 A), additional hard food pellets and water-softened food on the floor of the cage, as well as access to a water bottle and standard cage water supply. Animal’s weights were tracked regularly and extra i.p. saline and softened food or diet gel were provided to encourage weight gain and proper hydration when appropriate.

Animals were housed in the animal care facilities and maintained as approved by IACUC at University of Florida and University of South Carolina, and followed guidelines established by National Institutes of Health. Food and water were available ad libitum in the home cage. The room was maintained under standard 12-h light/dark cycles, at 22–24^o^C with 50–60% humidity.

#### MPTP administration

C57/BL6 mice underwent an acute MPTP injection paradigm (four doses every 2 h, i.p.)^165^. Mice were injected with MPTP hydrochloride (dissolved in 0.9% saline)(Sigma–Aldrich M0896), while control mice were administered an equal volume of saline (Hospira, Inc., Lake Forest, IL, USA). Male mice received a dose of 18 mg/kg MPTP. The lesions were allowed to stabilize seven days before additional drugs were administered. Following this stabilization period, mice were administered either L-Dopa (6.25 mg/kg, i.p, Sigma, D1507), a combination of L-Dopa (6.25 mg/kg, i.p.) and benserazide hydrochloride (10 mg/kg, i.p. Sigma, B7283), or saline alone once a day for three days. All compounds were dissolved in 0.9% saline.

We note that MPTP is a DAT substrate and can affect immune cells expressing DAT when injected peripherally (i.p.). Murine PBMCs have a half-life of 18–24 h, after which they are replenished from bone marrow^202,203^. MPTP, once administered, metabolizes to MPP+ and is fully eliminated within 96 h (four days) post injection. By 6 days post injection, there are no remaining effects of MPTP on peripheral immune cells. To address potential confounds arising from MPTP interactions with circulating immune cells expressing DAT, drug injections took place 6–9 days following MPTP injection, with flow cytometry experiments (see below) conducted on day 9.

#### 6-OHDA administration

Under isoflurane anesthesia, the mice were placed in a stereotaxic frame (David Kopf Instruments) and maintained throughout surgery using 1–2% isoflurane. Bilateral internal cannulas (Plastics One), for delivery of 6-OHDA to the median forebrain bundle, were positioned ±1.1 mm lateral and −5.0 mm ventral and were implanted 0.8 mm posterior to Bregma). 6-OHDA (Tocris 2547) was prepared at a concentration of 5 μg/μL in 0.9% NaCl containing 0.01% ascorbate (w/v) for bilateral depletions. Injections were performed using a 33-gauge cannula (Plastics One) attached to a 10 μL Hamilton syringe within a syringe pump running at 0.5 μL/min, to a total volume of 1 μL/side. The injection cannula was left in place for 5 min following the injection. Following recovery, beginning the day following surgery, mice were administered either L-Dopa (6.25 mg/kg, i.p, Sigma, D1507), a combination of L-Dopa (6.25 mg/kg, i.p.) and benserazide hydrochloride (10 mg/kg, i.p. Sigma, B7283), or saline alone once a day for 5 days. All compounds were dissolved in 0.9% saline.

### CPCA-TH Alexa647 Conjugation

Chicken anti-TH antibody (Encor, CPCA-TH) was conjugated to AlexaFluor 647 for use in flow cytometry as follows. 0.5 mL of 2.176 mg/mL antigen affinity purified chicken polyclonal antibody to tyrosine hydroxylase in PBS, derived from EnCor product CPCA-TH, was mixed with 50 microliters of 1 M bicarbonate buffer pH = 8.3. The mixture was then pipetted into one vial of Alexa Fluor 647 labelling reagent (Thermo-Fisher, A20173). Each vial contains a mini-stirrer bar so the reaction mixture was incubated for one hour at room temperature on a magnetic stirrer in the dark. The mixture was then applied to a Biorad 10DG gel filtration column (cat # 7322010) equilibrated in PBS and fractions containing conjugated antibody were pooled. The labeling level was calculated to be 4.72 fluorochromes per IgY molecule.

### PBMC isolation

#### Human

As previously published^20,21,69,123^, whole blood was collected in K2EDTA vacutainer blood collection tubes (BD, 366643) and kept at room temperature for up to 2 h prior to PBMC isolation. Briefly, blood from healthy volunteers and PD patients was overlaid in Leucosep tubes (Table 2) for PBMC isolation, centrifuged for 20 min at 400 g with brakes turned off and acceleration set to minimum. PBMCs were collected from the interphase of Ficoll and PBS, transferred to a fresh 15 mL conical tube, resuspended in 8 mL sterile PBS and centrifuged for 10 min at 100 × g, and repeated twice more. Cells were counted with a hemacytometer using trypan blue exclusion of dead cells, and density adjusted with PBS for flow cytometry staining.

#### Mouse

Whole blood was obtained *via* cardiac puncture when the animal was deeply anesthetized. Up to 1 mL whole blood collected in a 25-guage 1 mL syringe, pretreated with PBS containing 1% EDTA, was transferred to K2EDTA vacutainer tube and held for up to 30 min prior to PBMC isolation. Whole blood was transferred from collection tube into a 15 mL conical tube containing 1 mL sterile PBS (1:1 dilution in PBS) and overlaid atop 1 mL sterile Ficoll-Paque Plus (GE, 45-001-750) in 5 mL FACS tubes. Overlaid blood samples were centrifuged for 20 min at 400 *x* g with brakes off and acceleration set to minimum. PBMCs collected from the interphase of Ficoll and PBS were transferred to a fresh 5 mL FACS tube (Table 2), resuspended with 4 mL sterile PBS and centrifuged for 10 min at 100 g, and repeated twice more. Cells were counted with a hemacytometer using trypan blue exclusion of dead cells, and density adjusted with PBS, for flow cytometry staining.

### Flow cytometry

#### Human

Antibody concentrations, vendors and catalog numbers are shown in Table 1. Reagent details are shown in Table 2. Equipment can be found in Table 3. As previously published^20,21,69,123^, primary patient PBMCs and healthy control subject PBMCs were stained for flow cytometry analysis in 100uL staining volume containing 1 million cells per condition. Staining cocktails for individual samples were conducted in three staining tubes, with combinations as follows: MDSC markers (CD11b, CD14, CD15, CD16, HLA-DR) and DAT and TH. These combinations were selected to manage antibody cross reactivity and intolerance of some surface markers to cell permeabilization. Live-cell staining for surface markers (CD11b, CD14, CD15, CD16, HLA-DR) was performed on ice for 30 min, followed by washes, fixation and permeabilization (eBioscience, 88-842-00). Staining for intracellular epitopes of DAT and TH was performed at room temperature in permeabilization buffer (eBioscience, 88-842-00), followed by species-specific secondary antibodies (Table 1). We note that the flow cytometry method used to detect DAT and TH expressing cells does not allow for assessment of protein levels of these markers. Samples were resuspended in 500 µL PBS after the final wash. Data were collected within 2 h on BD Canto II, BD LSR II, or Sony Spectral Analyzer SP6800. Each experiment included single color compensation, followed by automatic compensation calculation. Compensation matrices were not altered thereafter. Data were analyzed using FCS Express 7 (De Novo Software), using analysis gates set by fluorescence minus one (FMO) analysis (Supplementary Fig. 1).

**Table 1:** Antibodies.

Specificity	Clone/species	Conjugate	Vendor	Catalog Number	Purpose	Dilution	Concentration
TH	Polyclonal/Rabbit	N/A	Sigma	AB152	FC	1:100	0.01 mg/mL
DAT	MAB369/Rat	N/A	Sigma	MAB369	FC	1:100	0.01 mg/mL
DAT	Polyclonal/Rabbit	N/A	Sigma	AB5802	FC	1:200	0.02 mg/mL
Rabbit	Polyclonal/Goat	BV421	BD	565014	FC	1:40	0.005 mg/mL
Rat	Polyclonal/Goat	APC	BD	551019	FC	1:40	0.005 mg/mL
CD14	MfP9/Mouse	FITC	BD	347493	FC	1:50	0.0005 mg/mL
CD14	M5E2/Mouse	PE	Biolegend	301805	FC	1:200	0.004 mg/mL
CD15	W6D3/Mouse	BV605	Biolegend	323031	FC	1:100	0.008 mg/mL
CD11b	ICRF44/Mouse	BV421	Biolegend	301323	FC	1:50	0.016 mg/mL
CD11b	M170/Rat	FITC	Biolegend	101205	FC	1:100	0.05 mg/mL
CD16	3G8/Mouse	APC	Biolegend	302011	FC	1:50	0.016 mg/mL
HLA-DR	L243/Mouse	APC-Cy7	Biolegend	307617	FC	1:50	0.016 mg/mL
Live/Dead	N/A	ZombieRed	Biolegend	423109	FC/Utility	1:1000	N/A
Ly6C	HK1.4/Rat	BV785	Biolegend	128041	FC	1:200	0.001 mg/mL
Ly6G	1A8/Rat	PE	Biolegend	127607	FC	1:100	0.002 mg/mL
TH	Polyclonal/Chicken	AF647	Encor	CPCA-TH	FC	1:45	0.01 mg/mL
TH	MCA-4H2/Mouse	N/A	Encor	MCA-4H2	IHC	1:500	0.002 mg/mL
IBA1	Polyclonal/Rabbit	N/A	Wako	019–19741	IHC	1:500	0.001 mg/mL
NET	Monoclonal/Mouse	N/A	MAB Tech	NET17–1	FC	1:100	0.005 mg/mL
Mouse	Polyclonal/Goat	HRP	Biolegend	405306	IHC	1:250	0.004 mg/mL
Rabbit	Polyclonal/Donkey	HRP	Biolegend	406401	IHC	1:250	0.004 mg/mL

**Table 2:** Reagents and materials.

Reagent	Supplier	Catalog number	Purpose	Concentration
Ficoll-Paque Plus	GE	45–001–750	PBMC isolation	N/A
PBS	In house	N/A	PBMC isolation, FC	1x
K2EDTA Vacutainer	BD	366643	Blood collection	N/A
Butterfly blood collection device	BD	367342	Blood collection	N/A
L-DOPA	Sigma	D1507	IP Injection (mouse)	6.25 mg/kg
Benserazide	Sigma	B7283	IP Injection (mouse)	10 mg/kg
FACS tubes	Fisher		FC, mouse PBMC isolation	N/A
6-OH-DA	Tocris	2547	IC injection (mouse)	5ug/uL
MPTP	Sigma	M0896	Injection (mouse)	
TritonX-100	ThermoFisher	BP151–100	IHC	0.5%
Goat serum	Lampire Biologicals	7332500	IHC	5%
Leucosep Tubes	Grenier BioOne	227290 P	PBMC isolation	N/A
Trypan Blue	MP Biomedicals	1691049	Cell counting	Stock
Fix/Perm Kit	eBioscience	88–8824–00	FC	Stock
Anti-TH	Encor	CPCA-TH	FC	1ug/mL
Leucosep Tube	Grenier BioOne	227290 P	PBMC isolation	N/A
Diet Gel	ClearH2O	76 A	Mouse diet supplement	Stock
Bilateral Cannula	P1	C235IS-5/SP	6-OH-DA infusion	N/A
Infusion tubing	P1	8F023X041P01	6-OH-DA infusion	N/A
Syringe	Exel	26016	IP injection, cardiac puncture blood draw	N/A
Isoflurane	Patterson	07–893–8441	Anesthesia	1–5%
Permount	Fisher	SP15–500	IHC	Stock
DAB substrate	Sigma	D4293	IHC	Stock
SG substrate	Vector Labs	VK4700	IHC	Stock
PFA	Sigma	158127	IHC	4%
AlexaFluor 647 labeling reagent	ThermoFisher	A20173	FC conjugation	Stock
Filtration column	Biorad	7322010	Affinity purification	N/A

**Table 3:** Equipment.

Equipment	Supplier	Part number	Purpose
Centrifuge	Sorvall	ST8	PBMC isolation
Cytometer	BD	Canto II	FC
Spectral Analyzer	Sony	SP6800	FC
Microcentrifuge	Fisher	59 A	FC
Dual syringe infusion pump	Harvard Apparatus		Surgery
Stereotax	Kopf	Model 940	Surgery
Cannula holder	Kopf	1776-P1	Surgery

#### Mouse

Antibody concentrations, vendor, catalog numbers and fluorochromes are shown in Table 1. Reagent details are shown in Table 2. Following counting and density adjustment, murine PBMCs were immediately fixed for 30 min (eBioscience, 88-842-00) protected from light, followed by washes and permeabilization. Surface and intracellular staining with antibodies against CD11b, Ly6C, Ly6G, DAT and TH was performed at room temperature, protected from light. Following washes, appropriate species-specific secondary antibodies were added and incubated for an additional 30 min. After final washes, samples were resuspended in 300 µL PBS. Data were immediately acquired on BD LSR II or Sony Spectral Analyzer SP6800. Each experiment included single color compensation, followed by automatic compensation calculation. Compensation matrices were not altered thereafter. Data were analyzed using FCS Express 7 (De Novo Software), using analysis gates set by FMO (Supplementary Fig. 8).

### Fluorescence minus one (FMO) analysis

Human and murine PBMC samples, stained as described above, were analyzed using gates set by fluorescence minus one (FMO). For each panel used, a separate set of samples was prepared in which a single fluorochrome was omitted per sample. After compensation, negative space created by omission of the fluorochrome was used to set positive gates. Set gates were verified using a fully stained sample. This procedure was repeated for each human panel (Supplementary Fig. 1) and murine panel (Supplementary Fig. 8). To maintain accuracy over time, FMOs were repeated monthly, when a new lot of fluorescent reagents was used, or when instrument settings were adjusted during routine servicing.

### Immunohistochemistry

Depletion of dopamine neurons in 6-OH-DA and MPTP-treated mice was confirmed using immunohistochemistry to assess midbrain TH+ neurons and microglial activation. Following terminal blood draw via cardiac puncture (see above), animals were perfused with 10 mL sterile PBS (pH 7.4) followed by 10 mL 4% paraformaldehyde (PFA) in PBS (pH 7.4). Brains were removed from the cranium and post-fixed in 4% PFA in PBS for 24 h at 4 ^o^C, then transferred to PBS containing 0.01% sodium azide. Fixed tissue was sectioned at 40um on a vibrating microtome. Midbrain sections were identified using the Paxinos & Franklin’s mouse brain atlas. Sections were quenched in 3% H_2_O_2_, twice for 10 min each, and blocked with 10% normal goat serum in PBS containing 0.3% TritonX-100 for 1 h at 37 ^o^C. Primary antibodies against TH (Encor, MCA-4H2) and microglial marker IBA1 (Wako, 019–19741) were added at a dilution of 1:500 and 1:800, respectively, and incubated overnight at room temperature. Following washes, HRP-conjugated anti-mouse secondary antibody was added and incubated for 1 h at room temperature, exposed to diaminobenzidine substrate for 10 min to produce a brown reaction product in TH+ neurons, then washed to remove excess antibody/substrate. A second HRP-conjugated secondary, anti-rabbit HRP, was added to detect IBA1+ microglia and incubated for 1 h at room temperature. Following washes, sections were incubated with Vector SG substrate (VK4700) for 10 min. Stained sections were dehydrated, mounted with Permount (Fisher, SP15–500) and imaged the following day as shown in Supplementary Fig. 7.

### Statistics

Data analyses were performed under blinded condition, by an individual who did not conduct the experiments. One-way ANOVA and Tukey’s correction for multiple comparisons was used when comparing three groups or more. Unpaired Student’s t-test (two-tailed) was used when comparing two groups. *P* < 0.05 was considered statistically significant. To assess normality, we applied D’Agostino–Pearson’s test for normality. Sex as a biological variable was assessed by separately analyzing human male and female subjects for each metric, as shown in Supplementary Figs. 5 & 6, using unpaired Student’s t-test (two-tailed) with Tukey’s correction for multiple comparisons. Multiple linear regression analyses were used to assess relationships between age, motor scores, treatment scores and outcome variables (DAT^+^ and TH^+^ PBMCs), with Bonferonni’s correction for multiple comparisons, alpha = 0.001 was considered significant. A matrix of scatter plots was used to qualitatively confirm non-collinearity between variables, with no significant interactions found (Supplementary Table 3). All statistical analyses were performed in GraphPad Prism 8 and SPSS.

## Supplementary information

Supplementary Figures and Tables

## Data Availability

All data are available upon reasonable request.
