# Supplementary material for: DAT and TH expression marks human Parkinson’s disease in peripheral immune cells
Source: NPJ Parkinsons Dis. 2022 Jun 7;8:72. doi: 10.1038/s41531-022-00333-8 (PMC9174333; doi:10.1038/s41531-022-00333-8)
Supplement: Supplementary file 1 — Supplementary Figures and Tables [file 41531_2022_333_MOESM1_ESM.pdf]

Supplemental Figure 1

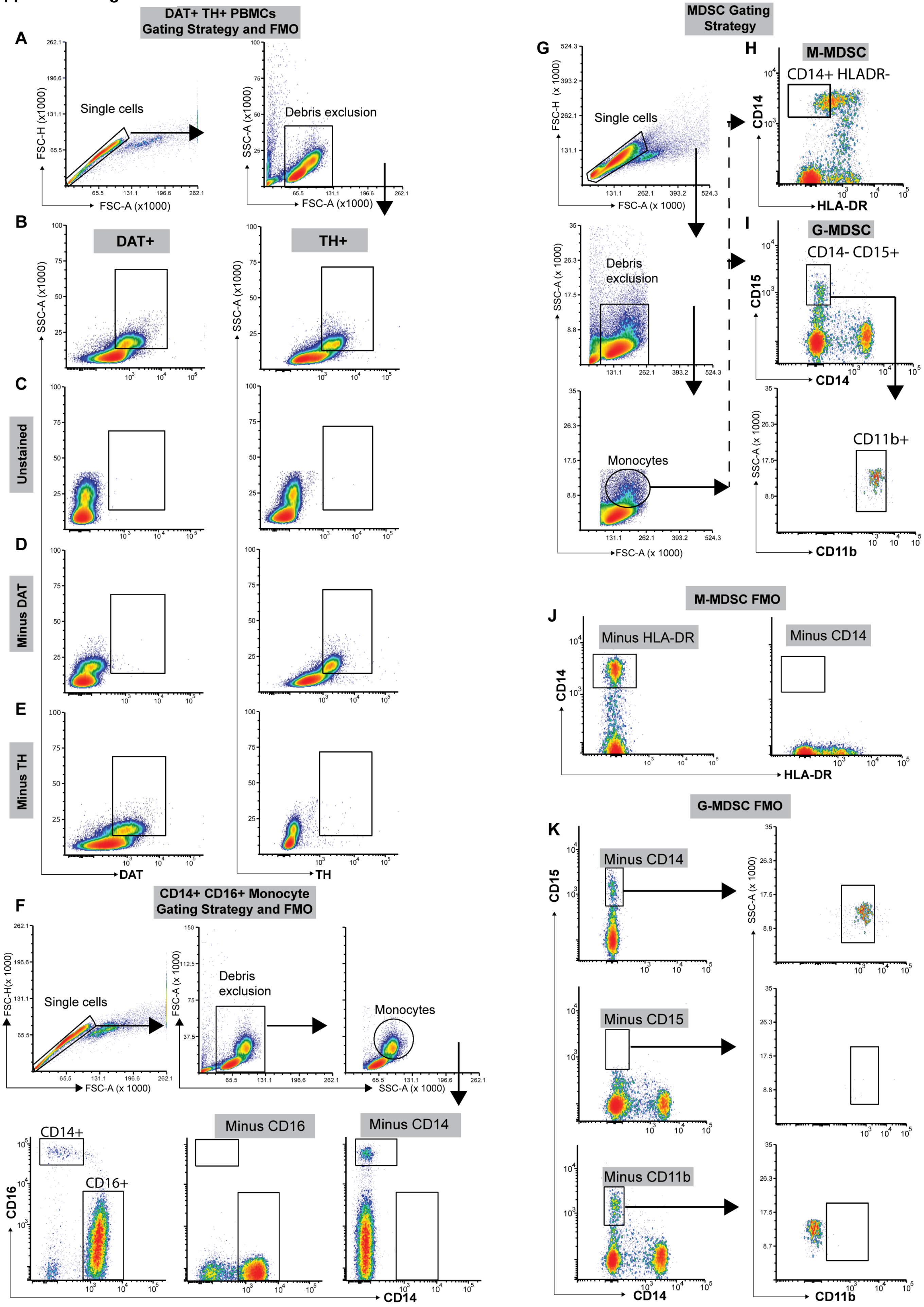

Supplemental Figure 1: Gating strategy and fluorescence minus one for TH and DAT expression in human monocytes.

Following isolation of single cells and exclusion of debris (A), gates for DAT^{+} and TH^{+} monocytes were set by fluorescence minus one (FMO). Unstained samples show no events in the gates set (B), while the minus-DAT condition shows TH staining but no DAT staining (C). The minus-TH condition shows DAT staining but no TH staining (D). The stained condition with both antibodies shows clear positive signals in both gates (E). For analysis of $CD14^{+}$ and $CD16^{+}$ monocytes, F) cell aggregates and debris were excluded, and monocytes gated by sidescatter were analyzed for expression of CD14 and CD16. (F, Lower panels): FMO analysis was used to set analysis gates for $CD14^{+}$ and $CD16^{+}$ monocytes. Monocytic MDSCS (M-MDSCs) and granulocytic MDSCs (G-MDSCs) were analyzed after excluding cell aggregates and debris. Monocytes gated by sidescatter were analyzed for H) CD14 expression and the absence of HLA-DR expression to define M-MDSCs, and monocytes expressing CD15 but not CD14 were analyzed for CD11b expression to define G-MDSCs . J-K) FMO analysis was used to set analysis gates for M-MDSCs and G-MDSCs as shown.

Supplemental Figure 2

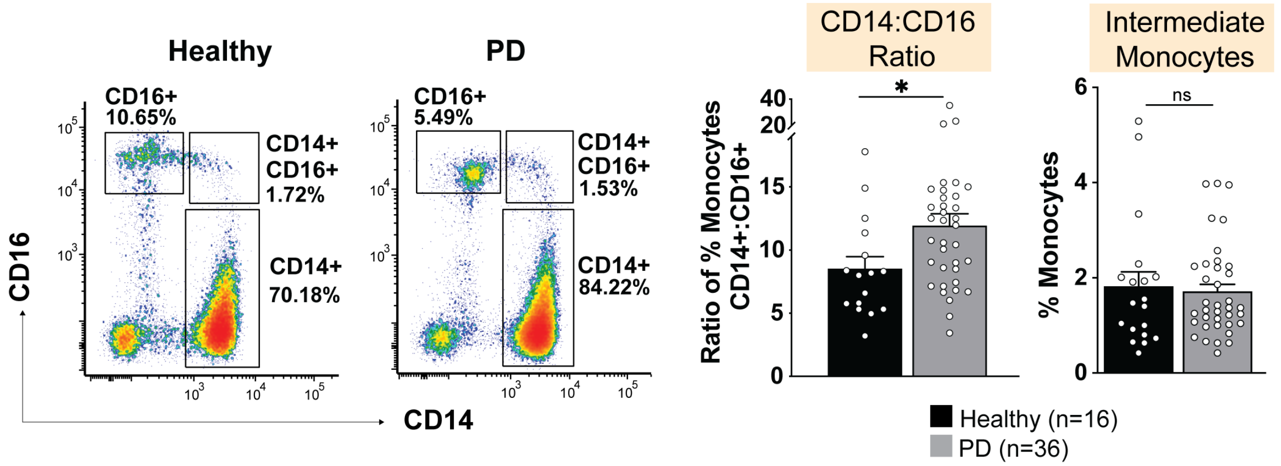

Supplemental Figure 2: PD patients and healthy controls do not exhibit significantly different populations of intermediate monocytes.

Monocytes from healthy controls (left) and PD patients (middle) defined by expression of CD14, CD16 or both. While PD patients show significant increase in the ratio of CD14:CD16 monocytes (middle-right), intermediate monocytes, defined as expressing both CD14 and CD16, are not significantly different between the two groups (far right). (Two-tailed Student's T-Test, with Tukey's post hoc; alpha=0.05)

Supplemental Figure 3

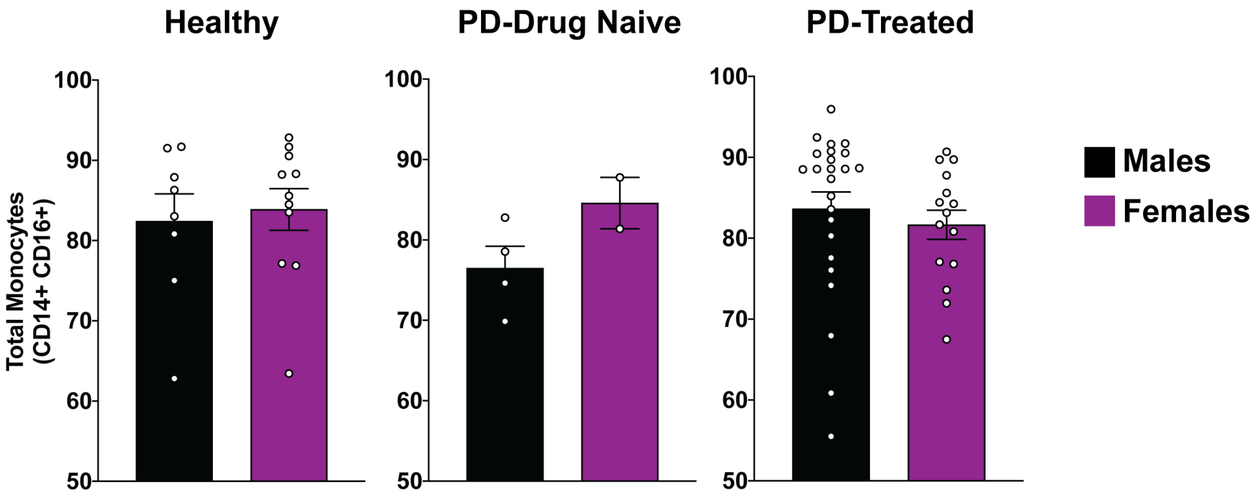

Supplemental Figure 3 : Sex-specific differences in total monocytes are not evident when comparing healthy controls, drug naïve and treated PD patients.

Healthy control subjects, drug naïve PD patients and treated PD patients do not exhibit significant differences in total numbers of CD14+CD16+ monocytes when assessing sex as a biological variable (two-tailed T-test, alpha=0.05, with Tukey's correction for multiple comparisons).

Supplemental Figure 4

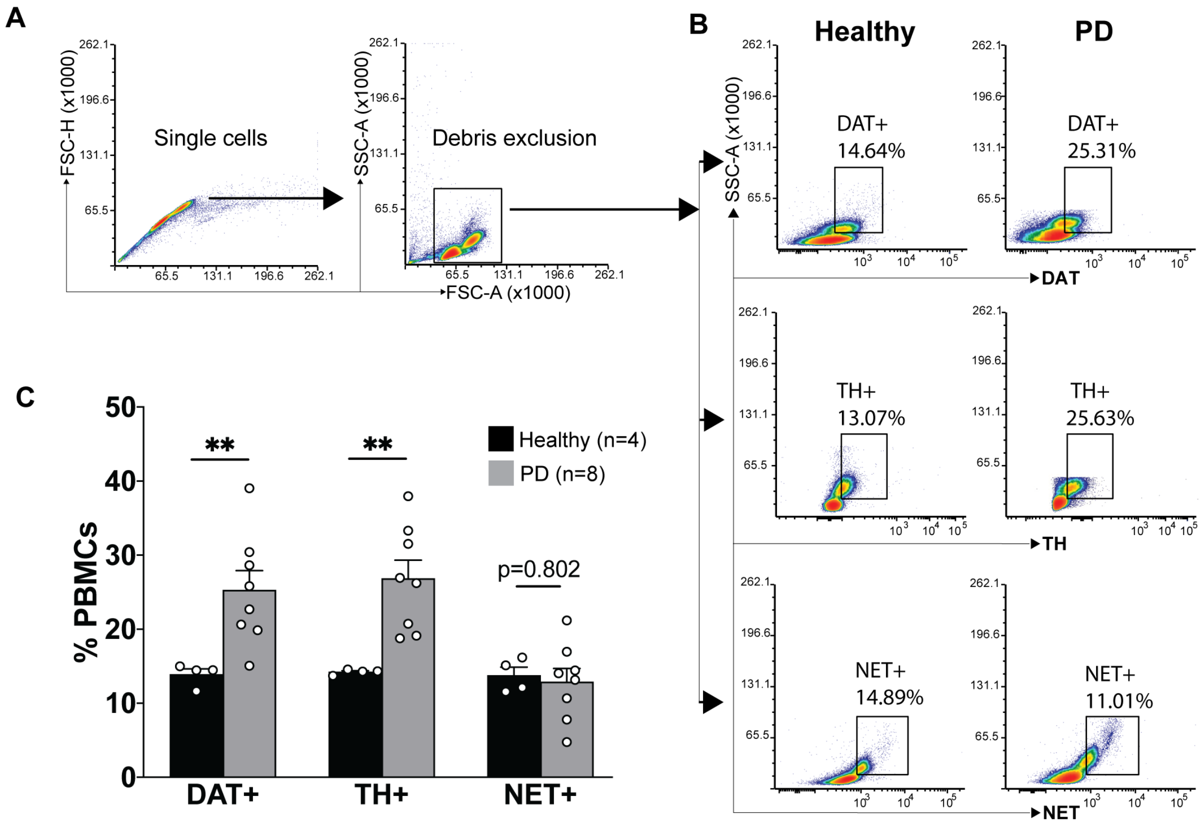

Supplemental Figure 4: Comparison of NET+ PBMCs does not show disease-associated increase, indicating specificity of increased DAT+ and TH+ PBMC in PD patients.

A) In a separate cohort of healthy control subjects (N=4) and Parkinson's disease patients (N=8), expression of norepinephrine transporter (NET) was assessed by flow cytometry and compared to DAT and TH expression in PBMCs. B) Representative plots showing that while DAT+ and TH+ PBMCs exhibit similar increases as shown in Figure 1-I, NET+ PBMCs do not change between the two groups. C) TH+ and DAT+ monocytes are significantly increased in PD patients relative to healthy controls ($P<0.01$, t-tests with Holm-Sidak correction for multiple comparisons, alpha=0.05, data shown as +SEM), while NET+ monocytes show no change in PD patients relative to healthy controls.

Supplemental Figure 5

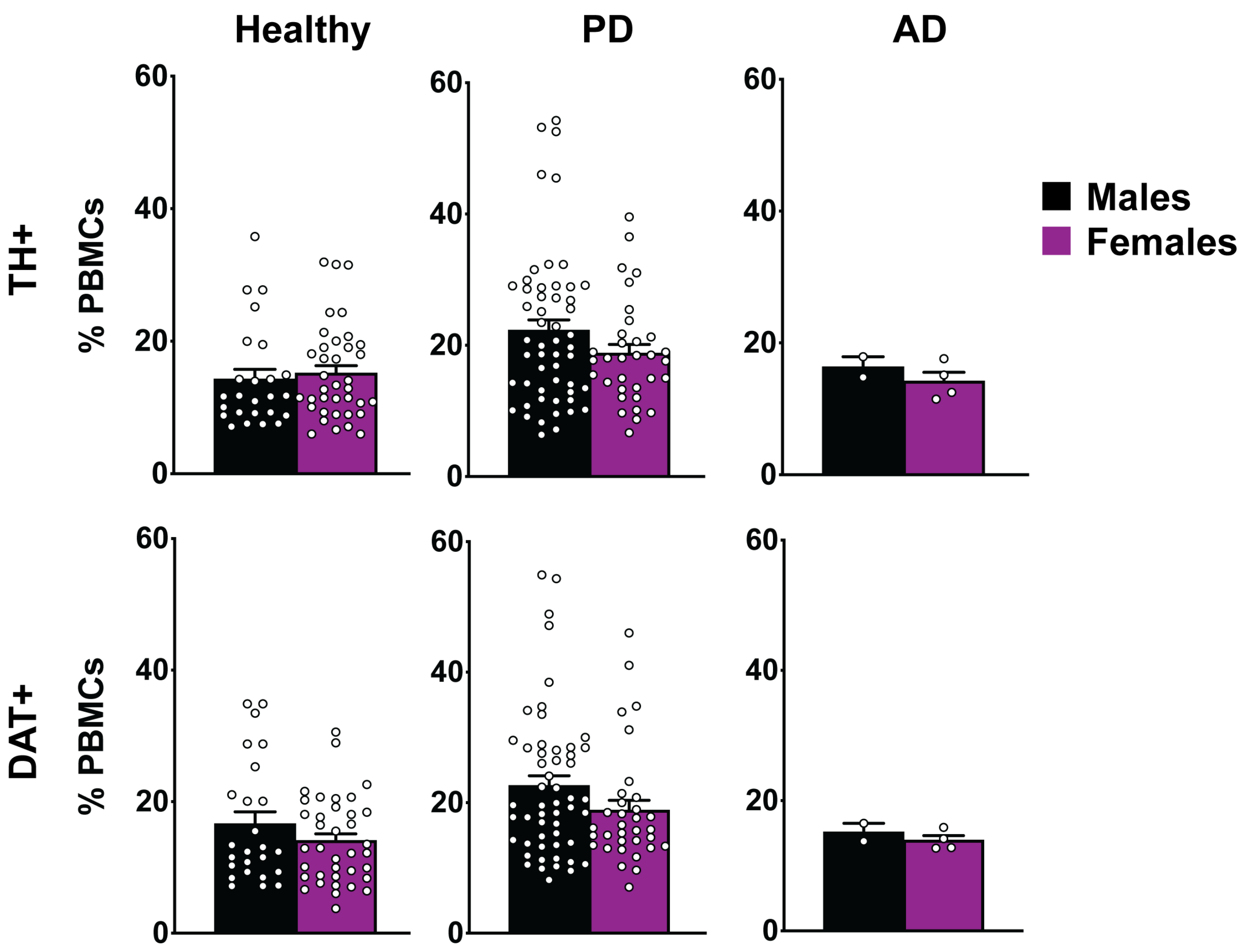

Supplemental Figure 5: Healthy, PD and AD subjects show no sex-specific difference in TH+ and DAT+ PBMCs. Data shown in Figure 1 were analyzed with females and males placed in separate analysis cohorts. No significant differences were observed between female and male subjects in the percent TH+ and DAT+ PBMCs. Data were analyzed by two-tailed T test, alpha=0.05.

Supplemental Figure 6

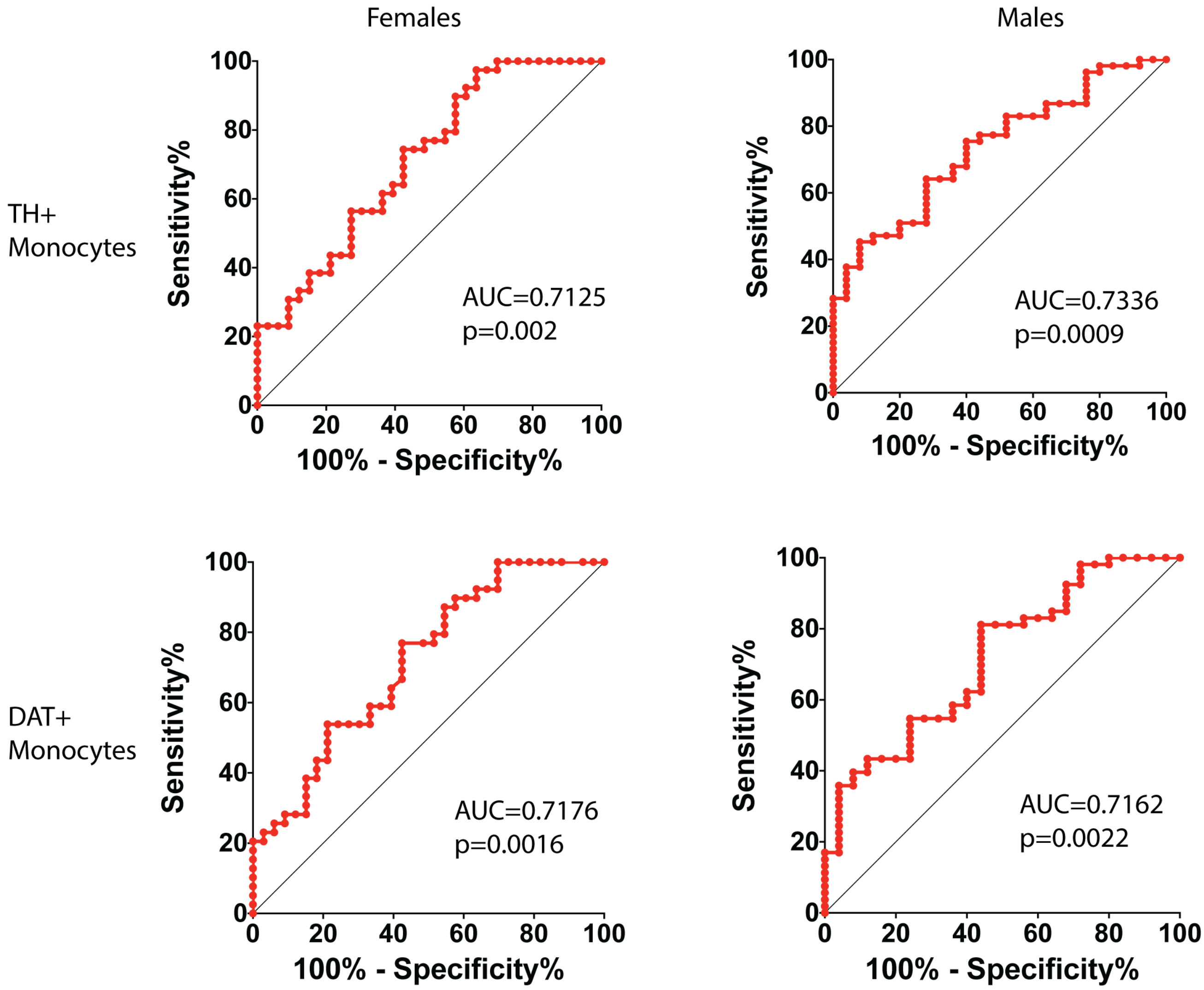

Supplemental Figure 6 ROC analysis shows similar trends when males and females are analyzed separately. AUC and significant values are similar to those obtained when analyzing combined male and female data, suggesting no clear gender when using DAT+ and TH+ PBMCs to classify PD patients and healthy control subjects.

Supplemental Figure 7

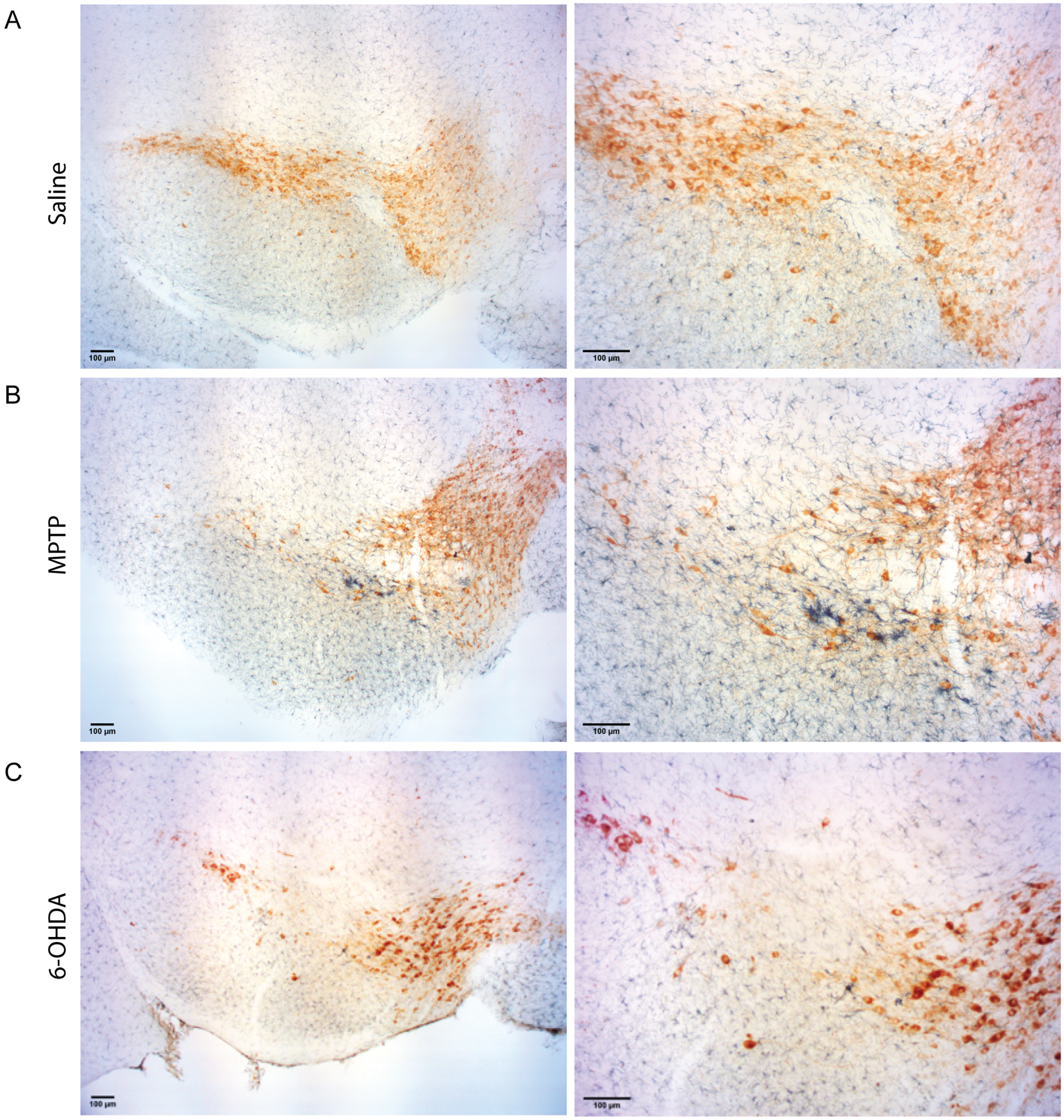

Supplemental Figure 7 MPTP and 6-OHDA mediated dopamine neuron depletion confirmed by decreased TH immunoreactivity in substantia nigra.

A-C) Representative images are shown from saline/sham treated, MPTP treated and 6OHDA treated murine midbrain sections. Clear lesion of substantia nigra dopamine neurons (identified by TH immunoreactivity, brown) are apparent in both lesioned conditions, and is associated with extensive microglial activation (identified by increased IBA1 immunoreactivity, grey/black).

Supplemental Figure 8

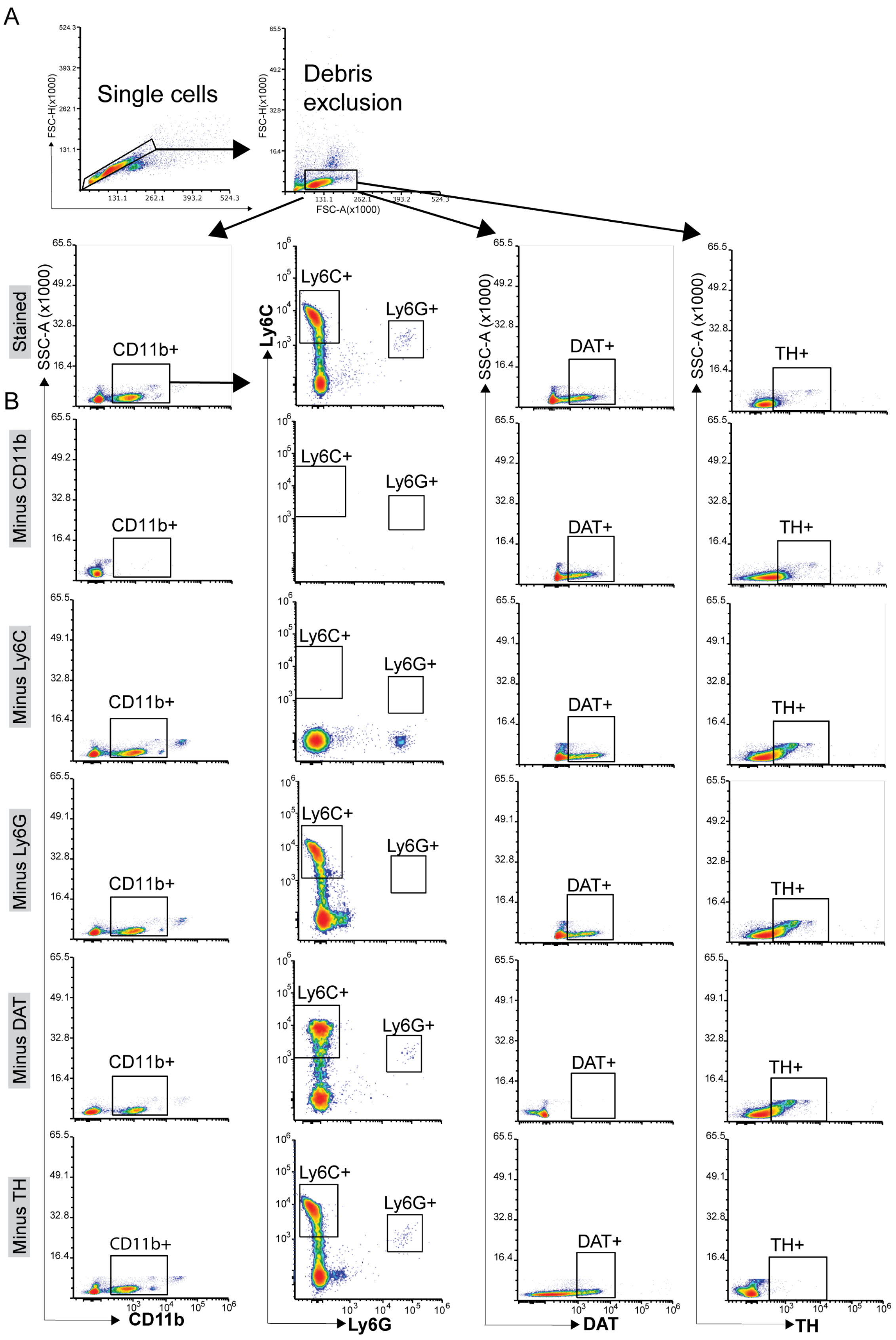

Supplemental Figure 8 Gating strategy and FMO for murine CD11b, Ly6C, Ly6G, DAT and TH expressing PBMCs.

A) After exclusion of cell aggregates and debris, PBMCs were analyzed for expression of CD11b, Ly6C, Ly6G, DAT and TH.
B) FMO for each condition were used to set analysis gates for each marker as shown.

Supplemental Figure 9

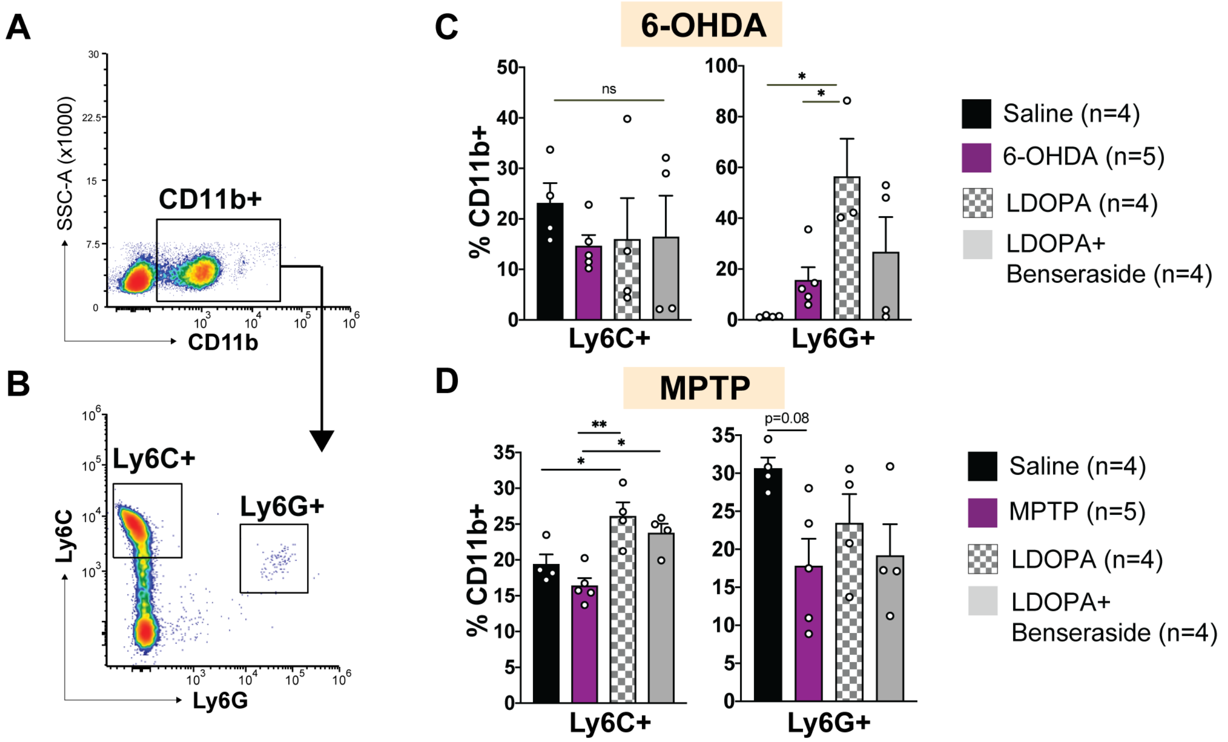

Supplemental Figure 9 In PD mice there is no increase in monocytic MDSCs.
A-B) Gating strategy for analysis of CD11b+ myeloid cells expressing Ly6C and Ly6G. C-D) Analysis of Ly6C+ monocytes (analogous to human M-MDSCs) and Ly6G+ monocytes (analogous to G-MDSCs) suggest that murine MDSC populations, in PD mice, do not mirror MDSCs in human PD patients, potentially due to the short time course of the lesion. Data are presented as +SEM, analyzed by one-way ANOVA, with Tukey's correction for multiple comparisons, alpha=0.05 (C-D).

Supplemental Figure 10

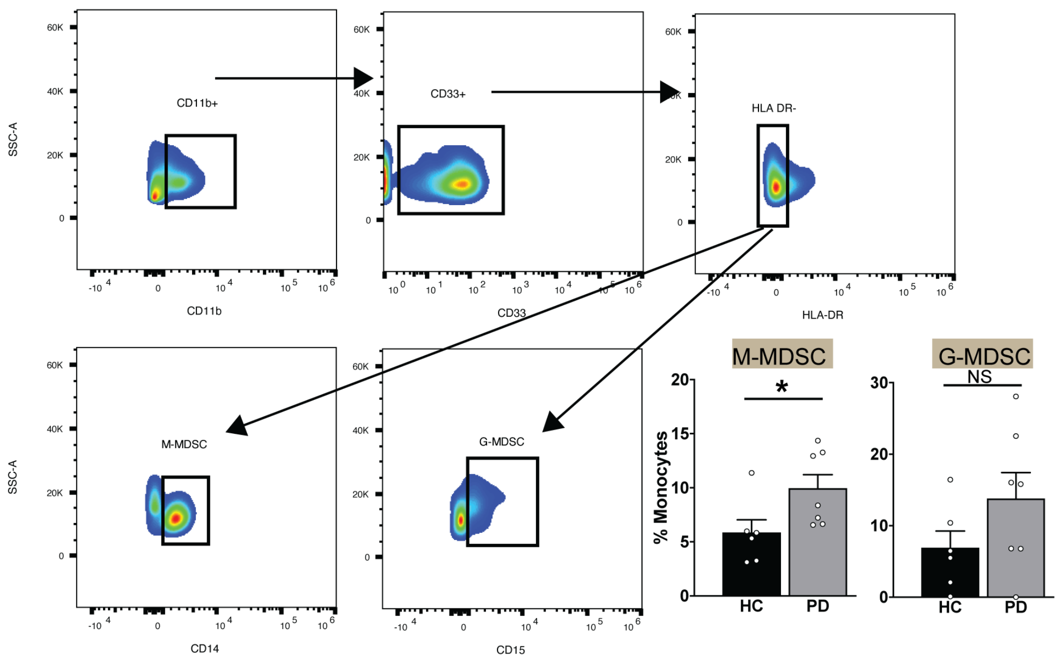

Supplemental Figure 10: Alternative MDSC gating strategy also reveals similarly increased M-MDSCs in PD, with no significant increase in G-MDSCs.

An additional gating strategy was used to analyze MDSC populations in PD patients and healthy controls, confirming increased M-MDSCs in PD patients compared to control subjects, with no significant change in G-MDSCs.

Supplementary Table 1: Clinical data for Parkinson's disease patients									
Patient ID	Disease Duration (Years)	Sex	Age (Years)	H-Y	UPDRS Off	UPDRS On	LED Score	Other Conditions	Medications
Parkinson's 4	9	F	70	--	46	33	906	Depression, Acid Reflux, Hypothyroidism	Amantadine, Elavil, Rytary, Famotidine, Neurontin, Robinul, Levothyroxine, Mobic, Aziltec
Parkinson's 5	--	M	76	2.5	33	29	1810	Chronic pain, Constipation	Tylenol, Sinemet CR, Sinemet, KlonoPIN, Cymbalta, Requip, Senokot, Ultracet
Parkinson's 6	--	M	63	2.00	43	25	300	Hypotension, Constipation	Sinemet, KlonoPIN, Proamatine, MiraLAX, Aziltec
Parkinson's 7	--	M	76	--	--	--	0	High Cholesterol, Hypertension, Hypothyroidism	Amoxil, Flexeril, Repatha, Flonase, Advil, Avapro, Levothyroxine, Pravachol, Mysoline, Inderal
Parkinson's 9	9	M	74	2.50	30	--	700	Restless leg syndrome	Aspirin, Sinemet CR, Sinemet, KlonoPIN, Aziltec, Neupro
Parkinson's 11	--	F	68	2.00	30	24	1835	B-12 deficiency Anemia	Sinemet CR, Sinemet, KlonoPIN, Colace, MiraLAX, Exelon, Cyanocobalamin, Requip, Eldepryl
Parkinson's 12	5	F	77	--	24	--	0	High Cholesterol, Hypertension, Acid Reflux	Tylenol, Lipitor, Coreg, Plavix, Prilosec
Parkinson's 13	--	F	46	--	--	--	1875	None	Sinemet CR, Sinemet, Artane
Parkinson's 14	14	M	69	3.00	24	13	1600	Anxiety, Insomnia	Sinemet, Flexeril, Valium, Paxil, Flomax, Restoril
Parkinson's 15	--	M	53	2.00	29	22	1325	None	Trihexyphenidyl, Sinemet CR, Sinemet, Amantadine, Entacapone
Parkinson's 16	8	M	75	2.50	30	38	950	High Cholesterol, Type II Diabetes, Angina, Acid Reflux, Hypertension	Aspirin, Sinemet CR, Sinemet, Aricept, Lescol, Amaryl, Metformin, Nitrostat, Aciphex, Cozaar, Toprol-XL
Parkinson's 17	8	M	74	2.00	--	15	750	None	Aspirin, Sinemet CR, Sinemet, Mirapex PO, Aziltec
Parkinson's 18	16	M	74	4.00	39	28	1075	High Cholesterol, Hypertension, Hypertriglyceridemia	Amantadine, Lipitor, Sinemet, KlonoPIN, Plavix, Lotrisone, Flonase, Lisinopril, Bactroban, Lovaza, Mirapex
Parkinson's 19	--	M	58	2.00	30	20	1300	Migraines, Depression	Amantadine, Fioricet, Lodosyn, Sinemet, KlonoPIN, Estrace, Norco, Inderal LA, Zoloft, Desyrel
Parkinson's 20	23	M	68	2.00	31	26	3000	None	Sinemet, KlonoPIN, Synalar, Nizoral, Ultram
Parkinson's 24	--	F	73	3.00	37	41	0	High Cholesterol, Type II Diabetes, Hypertension, Insomnia	Aspirin, Lipitor, KlonoPIN, Flexeril, Metformin, Lopressor, Ultram, Ambien
Parkinson's 25	8	M	69	--	16	15	500	Acid Reflux, Erectile dysfunction	Sinemet CR, Famotidine, Cialis
Parkinson's 26	4	F	78	1.50	24	11	300	Chronic heart failure, Constipation, Hypothyroidism	Sinemet, Colace, Hydrodiuril, Xalatan, Levothyroxine, Aleve PO
Parkinson's 27	21	M	71	3.00	--	32	300	Asthma, High Cholesterol, Depression, Hypertension	Proventil, Aspirin, Sinemet, Vytorin, Prozac, Lasix, Cozaar, Augmentin, Tessalon, Vibramycin, Deltasone
Parkinson's 28	--	M	62	2.00	17	8	2170	Angina, Depression, Hypertension	Tenormin, Sinemet, CeleXA, Benicar HCT, Requip
Parkinson's 29	4	F	77	--	13	--	0	Constipation, Overactive bladder, Depression	Colace, Estrace, Allegra, Flonase, Neurontin, Advil PO, Ditropan, Zoloft
Parkinson's 30	--	M	67	--	30	--	400	Hypertension, High Chlolesterol	Norvasc, Tenormin, Lipitor, Sinemet, Avapro, Aldactone, Artane

Supplementary Table 1: Clinical data for Parkinson's disease patients									
Patient ID	Disease Duration (Years)	Sex	Age (Years)	H-Y	UPDRS Off	UPDRS On	LED Score	Other Conditions	Medications
Parkinson's 31	--	F	61	2.50	29	28	1200	Hypothyroidism, Depression	Sinemet, KlonoPIN, Levothyroxine, Zoloft, Desyrel
Parkinson's 32	5	M	64	2.00	41	40	1200	Hypertension, Potassium deficiency, Constipation	Aspirin, Sinemet, Lasix, Lopressor, KLOR-CON M, Altace, Senokot
Parkinson's 33	22	F	60	4.00	28	24	2000	Overactive bladder, Hypothyroidism, Depression	Toviaz, Levothyroxine, Amantadine, Sinemet, Mirapex, Zoloft
Parkinson's 37	13	M	53	--	26	21	1750	Water retention/edema	Sinemet CR, Sinemet, Flonase, Hydrodiuril, Mirapex, Eldepryl
Parkinson's 38	13	M	63	2.00	--	22	1950	Depression	Acetylcysteine, Amantadine, B Complex Plus PO, Sinemet CR, Sinemet, Roxicodone, Mirapex, Zoloft
Parkinson's 39	--	M	69	4.00	46	37	1300	Constipation, Type II Diabetes, High Cholesterol, Migraines, Hypertriglyceridemia	Sinemet CR, KlonoPIN, Colace, Comtan, Neurontin, Glipizide XL, Mevacor, Metformin, Lovaza, Percocet, Mirapex, Fioricet
Parkinson's 40	9	M	77	--	--	24	400	Acid Reflux, exema, enlarged prostate, Hypertension	Tenormin, Sinemet, Proscar, Neurontin, Plaquenil, Prilosec, Deltasone, Aziltec, Flomax
Parkinson's 41	24	F	70	--	40	30	650	Hypothyroidism, Depression	Tylenol PO, Sinemet CR, Levothyroxine, Mirapex, Zoloft
Parkinson's 42	--	M	76	2.00	27	21	770	High Cholesterol,	Amantadine, Lipitor, Sinemet CR, Sinemet, Aricept, Xalatan, Roxicodone, Aziltec, Requip
Parkinson's 43	6	M	79	3.00	--	28	600	Psoriasis, Angina, Hypothyroidism, Hypertension, Acid Reflux, Enlarged prostate	Aspirin, Sinemet, Cimzia, Temovate, KlonoPIN, Neurontin, Imdur, Levothyroxine, Toprol XL, Nitrostat, Pravachol, Zantac, Flomax
Parkinson's 44	13	M	70	2.00	--	21	1600	None	Sinemet, Aziltec, Prevident 500 PLUS DT
Parkinson's 45	7	M	69	2.00	31	28	800	Asthma, High Cholesterol	Sinemet, KlonoPIN, Dulera, Chlolestoff, Mirapex
Parkinson's 46	15	M	68	3.00	44	36	4650	Depression, IBS, Constipation, Eczema/Psoriasis, Hypothyrodism, Overactive bladder, Acid Reflux	Aspirin, Biotin Max Strength, Wellbutrin XL, Sinemet CR, Sinemet, SB Docusate, Loprox, CeleXA, KlonoPIN, Desowen, Bentyl, Colace, Synalar, Nizoral, Levothyroxine, Myrbetriq, Protonix
Parkinson's 47	6	F	66	2.00	19	--	0	None	--
Parkinson's 48	23	M	65	4.00	--	36	1950	High Cholesterol, Enlarged prostate, Hypotension, Asthma, Overactive bladder, Depression	Aspirin, Lipitor, Super B Complex PO, Duopa Enteral, Sinemet CR, Sinemet, KlonoPIN, Proscar, Proamatine, Singulair, Ditropan, Zoloft
Parkinson's 49	--	M	53	1.50	--	8	700	Asthma, Hypothyroidsim, Hypertension, Acid Reflux, High Cholesterol,	Amantadine, Sinemet, KlonoPIN, Flonase, Advair, Levothyroxine, Bystolic, EQ Omeprazole, Zocor, Synthroid
Parkinson's 52	16	F	57	2.00	32	--	0	Depression, Pseudobulbar affect	Parcopa, Venlafaxine, Trazadone, Neudextra
Parkinson's 53	19	M	65	2.00	--	33	1050	Erectile dysfunction, Arthritis, Type II Diabetes, Enlarged prostate, High Cholesterol	Aspirin, B-Complex PO, Sinemet, Voltaren, Comtan, Metformin, Mirapex, Saw Palmetto PO, Zocor, Ultram, Antihistamine PO, Viagra

Supplementary Table 1: Clinical data for Parkinson’s disease patients									
Patient ID	Disease Duration (Years)	Sex	Age (Years)	H-Y	UPDRS Off	UPDRS On	LED Score	Other Conditions	Medications
Parkinson’s 54	9	M	74	2.50	--	16	750	Hypothyroidism, Acid Reflux, Enlarged prostate	Aspirin, Sinemet CR, Sinemet, Neurontin, Levothyroxine, Prilosec, Flomax
Parkinson’s 56	--	F	59	--	29	24	1000	High Cholesterol, Hypertension, Angina	Aspirin, Lipitor, Sinemet, KlonoPIN, Metformin, Toprol-XL, Roxicodone
Parkinson’s 57	--	M	77	2.00	--	23	400	Herpes, Depression, Hypertension, High Cholesterol, Sleep apnea, Acid Reflux	Zovirax, Aspirin, Sinemet, KlonoPIN, Benadryl, Prozac, Hydrodiuril, Zaditor, Cozaar, Mevacor, Lopressor, Provigil, Prilosec
Parkinson’s 58	10	M	68	--	37	--	300	High Cholesterol	Amantadine, Lipitor, Mirapex, Percocet, Aziltec
Parkinson’s 59	18	F	67	2.00	--	16	900	Depression, Arthritis	Tylenol, Amantadine, Sinemet CR, Sinemet, Zyrtec, Cymbalta, Flonase, Advil, Mobic
Parkinson’s 60	11	F	45	2.00	25	--	700	None	Amantadine, Sinemet, Blisovi
Parkinson’s 62	17	F	67	5.00	60	31	1060	Depression, Type II Diabetes, Acid Reflux, Arthritis	Amantadine, Aspirin, Sinemet CR, Zyrtec, KlonoPIN, Voltaren, Cymbalta, Neurontin, Glucotrol XL, Cozaar, Glumetza, Toprol-XL, Prilosec, Requip XL
Parkinson’s 64	20	F	65	--	36	21	230	Hypothyroidism, Depression	Sinemet CR, Tranxene, Flexeril, Benadryl, Levothyroxine, Remeron, Requip
Parkinson’s 65	4	M	74	2.00	17	--	0	High Cholesterol, Hypertension, Migraines, Type II Diabetes, Acid Reflux	Tylenol, Aspirin, Lipitor, Cardizem CD, Relpax, Jardiance, Prilosec
Parkinson’s 66	20	M	71	--	45	26	1400	Herpes, Hypertension, Depression, High Cholesterol, Erectile dysfunction	Zovirax, Sinemet, Cozaar, Roxicodone, Seroquel, Crestor, Viagra
Parkinson’s 67	--	M	76	2.00	33	26	1350	Constipation, Enlarged prostate, Conjunctivitis, Hypotension, Acid Reflux, B-12 deficiency	Tylenol, Sinemet CR, Sinemet, Colace, Aricept, Romycin, Proscar, Flonase, Nizoral, Proamatine, Aleve, Prilosec, Cyanocobalamin
Parkinson’s 68	15	M	57	2.50	37	27	1800	Type II Diabetes, Arthritis, Depression, Enlarged prostate, Erectile dysfunction,	Sinemet, Depakote ER, Comtan, Amaryl, Advil, Indocin, Nizoral, Remeron, Mirapex, Flomax, Tobradex, Staxyn, Effexor, Sinemet CR, Metformin, Cialis, Ultram
Parkinson’s 69	--	F	77	--	--	--	1735	Constipation, B-12 deficiency	Sinemet CR, Sinemet, KlonoPIN, Colace, MiraLAX, Exelon, Cyanocobalamin, Roxicodone, Requip, Eldepryl
Parkinson’s 71	4	M	64	3.00	--	30	1500	High Cholesterol, Hypertension, Anxiety, Hypertriglyceridemia, Potassium deficiency	Lipitor, Sinemet, Neurontin, Hydrodiuril, Atarax, Claritin, Toprol-XL, Lovaza, KLOR-CON M
Parkinson’s 72	--	F	66	2.00	22	--	40	None	Requip
Parkinson’s 73	14	M	72	2.00	14	--	1820	Depression, High Cholesterol, Hypertriglyceridemia, Hypertension	Ropinirole XR, Sinemet, Aspirin, Duloxetine, Fenofibrate, Tramadol, Valsartan
Parkinson’s 74	3	M	74	--	15	--	0	None	Aspirin
Parkinson’s	3	M	71	1.50	26	--	0	None	--

Supplementary Table 1: Clinical data for Parkinson's disease patients									
Patient ID	Disease Duration (Years)	Sex	Age (Years)	H-Y	UPDRS Off	UPDRS On	LED Score	Other Conditions	Medications
75									
Parkinson's 76	--	F	78	3.00	--	36	1000	Arthritis, High Cholesterol, Anxiety, Constipation	Aspirin, Sinemet, Voltaren, Vivelle-DOT, Tricor, Zymaxid, Atarax, Advil, Avapro, Acular, Mycolog II, MiraLAX, Aziltec, Exelon, Zocor
Parkinson's 78	19	M	72	--	32	--	600	Hypotension, Acid Reflux, Eczema, Enlarged prostate, Depression	Sinemet, Clomid, KlonoPIN, Northera, Neurontin, Plaquenil, Nizoral, Prilosec, Deltasone, Flomax, Effexor-XR
Parkinson's 80	22	M	58	--	40	51	600	Erectile dysfunction, Depression, Overactive bladder, High Cholesterol, Insomnia	Amantadine, Sinemet, Lexapro, Namenda, Ditropan, Zocor, Restoril, Viagra
Parkinson's 81	--	M	71	2.00	22	17	2394	Depression, Acid Reflux, High Cholesterol	Amoxil, Aspirin, Stalevo, KlonoPIN, Prozac, Neurontin, Robinul, Antivert, Zocor
Parkinson's 82	--	M	61	--	--	--	0	Arthritis	Zyrtec, Celebrex, Keflex, KlonoPIN, Bactroban, Roxicodone
Parkinson's 83	--	M	74	2.50	--	34	950	Enlarged prostate, High Cholesterol, congestive heart failure, Enlarged prostate, Hypothyroidism, Hypertension, Asthma, Myasthenia gravis	Uroxatral, Aspirin, Lipitor, Bumex, Rocaltrol, Sinemet CR, Sinemet, Proscar, Neurontin, Norco, Levothyroxine, Lopressor, Singulair, Metamucil, Mestinon
Parkinson's 84	18	F	63	--	20	--	1200	None	Aspirin, Astelin, Sinemet, Skelaxin, Nasonex, Aleve, Flumadine
Parkinson's 85	--	M	75	3.00	29	--	1125	Depression	Sinemet CR, Celexa
Parkinson's 86	--	M	58	2.00	13	--	405	High Cholesterol	Aspirin, Lipitor, Rytary ER, Rytary, Zofran
Parkinson's 88	11	F	76	4.00	43	39	--	High Cholesterol	Aspirin, Lipitor, Sinemet
Parkinson's 89	--	F	81	3.00	42	--	0	Anxiety, Hypertriglyceridemia	Xanax, Aspirin, Buspar, Allegra, Lovaza
Parkinson's 90	--	M	59	--	--	30	1600	Depression	Aspirin, Wellbutrin XL, Sinemet, Lexapro, Flonase, Advil, Skelaxin
Parkinson's 92	--	M	76	2.50	39	24	1250	Hypertension, Acid Reflux, Atril Fibrillation, Insomnia, High Cholesterol, Migraines, RLS	Tenormin, Fioricet, Sinemet, Zyrtec, KlonoPIN, Nexium, Cozaar, Xarelto, Neupro, Zoloft, Zocor, Ultram
Parkinson's 93	--	M	73	4.00	51	--	0	Hypertension, High Cholesterol, Constipation, Type II Diabetes, Insomnia	Norvasc, Lipitor, KlonoPIN, Flexeril, Colace, Hydrodiuril, Advil, Metformin, Lopressor, Ultram, Ambien
Parkinson's 95	11	M	64	--	--	--	1000	Depression, Constipation, Hypertension	Amantadine, Wellbutrin XL, Sinemet, Colace, Microzide, Lisinopril, Zocor, Coumadin
Parkinson's 96	--	F	70	4.00	--	41	3300	Depression, Overactive bladder	Sinemet CR, Sinemet, KlonoPIN, Claritin, Mirapex, Zoloft, Vesicare
Parkinson's 97	11	M	70	--	--	35	1050	High Cholesterol, Hypertension	Lipitor, Sinemet CR, Sinemet, Mirapex, Altace, Exelon
Parkinson's 98	11	F	72	2.00	19	16	1800	High Cholesterol, Depression, Hypothyroidism	Amantadine, Lipitor, Sinemet, Cymbalta, Levothyroxine, Ultram
Parkinson's 100	3	F	63	2.00	--	19	400	Anxiety, Hypertension, Hypothyroidism, Asthma, High Cholesterol	Aspirin, Buspar, Coreg, Zyrtec, Levothyroxine, Singulair, Altace, Zocor, Kenalog, Sinemet

Supplementary Table 1: Clinical data for Parkinson's disease patients									
Patient ID	Disease Duration (Years)	Sex	Age (Years)	H-Y	UPDRS Off	UPDRS On	LED Score	Other Conditions	Medications
Parkinson's 101	--	F	66	3.00	50	28	1900	Migraines	Amantadine, Super B Complex PO, Fioricet, Sinemet, MAG-OX, Roxicodone, Requip
Parkinson's 102	25	M	73	2.00	--	27	1000	Enlarged prostate, Type II Diabetes, Hypertension, Overactive bladder	Sinemet, Cleocin, Aricept, Proscar, Metformin, Toprol XL, Ditropan
Parkinson's 103	--	M	77	--	--	--	400	Kidney stones, Hypertension, Depression, Hyperparathyroidism, Hypertriglyceridemia, Acid Reflux, Enlarged prostate	Zyloprim, Norvasc, Aspirin, Astelin, Wellbutrin XL, Tegretol, Sinemet, Sensipar, Temovate, Lisinopril, Lopressor, Lovaza, Prilosec, Zemplar, Seroquel, Zoloft, Flomax, Desyrel
Parkinson's 104	11	M	71	2.00	--	29	1000	Restless leg syndrome	Rytary, Neupro
Parkinson's 105	6	M	60	--	--	12	100	Restless leg syndrome	Sinemet, Neupro, Artane
Parkinson's 107	24	F	73	3.00	--	35	2050	Osteoporosis, Constipation, Depression	Fosamax, Amantadine, Sinemet CR, Sinemet, KlonoPIN, DDAVP, Lidex, MiraLAX, Aziltec, Zoloft
Parkinson's 108	11	M	71	4.00	40	--	2100	Enlarged prostate, Addison Disease, Hypotension, Fibromyalgia, Depression, Erectile dysfunction	Uroxatral, Amantadine, Sinemet CR, Sinemet, KlonoPIN, Flexeril, Florinef, Acular, Proamatine, Lyrica, Effexor-XR, Viagra
Parkinson's 109	16	F	75	2.00	16	14	1600	Hypothyroidism, Depression, Insomnia	Sinemet, Levothyroxine, Zoloft, Restoril, Bactrim
Parkinson's 110	--	M	56	3.00	--	36	171	RLS	Rytary, Neupro
Parkinson's 111		M	58	2.00	--	17	25000	RLS, Arthritis, Herpes, Depression, Acid Reflux, Constipation, Ulcerative colitis, Arthritis, Kidney disease	Orencia, Zovirax, Apokyn, Sinemet, Celexa, KlonoPIN, Prilosec OTC, Metamucil, Azulfidine, Flexeril, Decadron, Ansaid, Neupro, Tigan
Parkinson's 112	15	F	66	1.50	18	15	819	Migraines, Depression	Fioricet, Stalevo, KlonoPIN, Flonase, Vimpat, Kepra, Aziltec, Requip XL, Zoloft
Parkinson's 113	--	F	69	3.00	41	33	1800	Hypothyroidism, Hypertension, High Cholesterol, Insomnia, Depression, B-12 deficiency	Aspirin, Sinemet CR, Sinemet, Levothyroxine, Proamatine, Macrobid, Mirapex, Crestor, Restoril, Effexor-XR, Cyanobalamin
Parkinson's 114	--	M	--	--	--	--	0	None	--
Parkinson's 118	--	M	--	--	--	--	0	None	--
Parkinson's 121	2	F	77	--	31	--	0	Hypertension, Constipation, Hypothyroidism, High Cholesterol	Norvasc, Aspirin, Colace, Levothyroxine, Lisinopril, MAG-OX, MiraLAX, Crestor
Parkinson's 123	--	M	60	--	--	--	1300	None	Amantadine, Sinemet, Mirapex
Parkinson's 124	14	M	64	2.50	21	--	600	RLS	Amantadine, Sinemet, Neurontin, Neupro
Parkinson's 125	2	F	--	--	--	--	100	None	Aspirin, DHA Complete PO, Aziltec
Parkinson's 126	3	M	70	2.00	15	12	0	None	--
Parkinson's 127	--	M	57	1.00	14	8	0	None	--
Parkinson's 128	3	F	70	1.00	10	--	0	None	--
Parkinson's 131	3	M	50	2.00	--	26	0	None	--

Supplementary Table 1: Clinical data for Parkinson's disease patients									
Patient ID	Disease Duration (Years)	Sex	Age (Years)	H-Y	UPDRS Off	UPDRS On	LED Score	Other Conditions	Medications
Parkinson's 132	21	F	70	2.00	38	37	1400	None	Sinemet, Pramipexole, Sinemet CR
Parkinson's 133	13	M	67	2.00	10	5	500	None	Sinemet, Sinemet CR, Aziltec
Parkinson's 134	21	F	71	2.50	--	29	700	None	Sinemet
Parkinson's 135	4	F	77	2.00	--	20	0	None	Rasagiline
Parkinson's 138	--	M	--	--	--	--	0	None	--
Parkinson's 139		M	--	--	--	--	--	None	--
Parkinson's 144	21	M	75	2.00	38	25	650	None	Levodopa, Levodopa CR
Parkinson's 147	3	M	55	1.00	28	15	0	None	--
Parkinson's 148		M	--	--	--	--	0	None	--

Supplemental Table 2- Statistical comparisons for age, sex, disease duration, gender distribution, motor scores, and LED for each group.

Figure Panels	# of patients	Age (Mean ± SD)	Age Range (Years)	# Male	# Female	Years Disease Duration (Mean ± SD)	Disease Duration Range (Years)	HY (Mean ±SD)	HY Range	UPDRS (Mean ± SD)	UPDRS range	LED (Mean ±SD)	LED Range
1C	36	67.61 (±7.95)	45-81	23	13	12.66 (±6.86)	0-23	2.48 (±0.83)	1.5-5	30.52 (±11.56)	13-60	1080.3 (±905)	0-4650
1E & 1G	9	66.33 (±7.39)	57-77	6	3	5.66 (±6.15)	0-14	2 (±1.22)	1-2.5	20.2 (±8.41)	10-31	500 (±594.41)	0-1300
2D	45	68.42 (±7.99)	45-81	29	16	10.93 (±8.11)	0-25	2.48 (±0.85)	1-5	31.26 (±12.97)	10-60	969.97 (±755.91)	0-3300
2H, I & J	96	68.03 (+7.55)	45-81	63	33	10.65 (±7.37)	0-25	2.52 (±0.77)	1.5-5	30.61 (±10.76)	13-60	1025.17 (±832.37)	0-4650
Statistical comparison* within cohort for each metric		n.s.				n.s.		n.s.		n.s.		n.s.	

*One-way ANOVA with Tukey's post-hoc

Supplemental Table 3: Statistical comparisons for age, disease duration, gender distribution, motor scores, and LED versus DAT+ and TH+ PBMCs. DAT+ and TH+ PBMCs were assessed for interactions with age, LED score, H-Y Score, UPDRS III scores, and disease duration via multiple linear regression. A matrix of scatter plots were used to assess multicollinearity. No significant interactions were found. Alpha=0.001 after Bonferonni's correction for multiple comparisons.

		R^2	P value	Significant?
**DAT+ PBMC vs LED**	*N=96 per group*	0.007	0.363	n.s.
**TH+ PBMC vs LED**		0.0091	0.451	n.s.
**DAT+ PBMC vs Age**		0.0558	0.1168	n.s.
**TH+ PBMC vs Age**		0.0273	0.196	n.s.
**DAT+ PBMC vs H-Y**		0.0011	0.339	n.s.
**TH+ PBMC vs H-Y**		0.0144	0.303	n.s.
**DAT+ PBMC vs UPDRS**		0.001	0.7509	n.s.
**TH+ PBMC vs UPDRS**		0.002	0.7189	n.s.
**DAT+ PBMC vs Disease Duration**		0.0114	0.3925	n.s.
**TH+ PBMC vs Disease Duration**		0.0114	0.3925	n.s.
